# Molecular correlates of sleep deprivation in the mouse brain identified by meta-analysis of microarray data

**DOI:** 10.1016/j.nbscr.2026.100149

**Published:** 2026-06-23

**Authors:** Osama H.M.H. Abdalla, Ella Dunlop, Tatiana S. Wilson, Paul K. Reardon, Mudassar Iqbal, Shu K.E. Tam, Vladyslav V. Vyazovskiy, David W. Ray, Laurence A. Brown, Hai-Ying Mary Cheng, Stuart N. Peirson

**Affiliations:** a Department of Biology, University of Toronto Mississauga, Mississauga, ON, L5L 1C6, Canada; b Department of Cell & Systems Biology, University of Toronto, Toronto, ON, M5S 3G5, Canada; c Sleep and Circadian Neuroscience Institute (SCNi), Kavli Institute of Nanoscience Discovery, Nuffield Department of Clinical Neurosciences, University of Oxford, United Kingdom; d Sleep and Circadian Neuroscience Institute (SCNi), NIHR Oxford Health Biomedical Research Centre, John Radcliffe Hospital, Oxford, OX3 9DU, United Kingdom; e Oxford Centre for Diabetes, Endocrinology and Metabolism, and Oxford Kavli Centre for Nanoscience Discovery, University of Oxford, Oxford, OX37LE, United Kingdom; f Section on Developmental Neurogenomics, Human Genetics Branch, National Institute of Mental Health, Bethesda, MD, 20892, USA; g Division of Informatics, Imaging & Data Sciences, University of Manchester, United Kingdom; h Duke Kunshan University (DKU), Kunshan, Jiangsu, China; i Sleep and Circadian Neuroscience Institute (SCNi), Kavli Institute of Nanoscience Discovery, Department of Physiology, Anatomy and Genetics, University of Oxford, United Kingdom; j Research IT, IT Services, University of Oxford, United Kingdom

**Keywords:** Meta-analysis, Sleep deprivation, *Rasd1*

## Abstract

Studying the transcriptional changes in the brain following sleep deprivation has provided insight into the molecular mechanisms that differ between sleep and wake. Individual studies are limited in their ability to detect differentially expressed genes due to small sample size. Here we performed a meta-analysis of published brain expression data, totalling 173 microarrays across 245 mice. 498 genes were identified as significantly changing with sleep-deprivation at q < 0.01, 96 of which were previously identified by the original studies. Of the remaining 402 novel candidate sleep genes, 14 were associated with human sleep traits and 3 with sleep phenotypes in knockout mice. Candidate gene validation showed significant upregulation of *Rasd1* (*Dexras1*) following sleep deprivation, and phenotyping of *Rasd1* KO mice revealed changes in the amount and distribution of behavioural sleep duration and sleep bout structure. These results provide a greater understanding of the molecular correlates of sleep and provide a resource for the sleep research community.

## Highlights

• Meta-Analysis of 173 microarrays identifies 498 genes linked to sleep deprivation.
• 402 of these genes were novel and not identified by the original studies.
• Pathways enriched among these genes: circadian, UPR, mRNA processing.
• QPCR validation highlights *Rasd1* upregulation after sleep deprivation.
• *Rasd1* KO mice show altered behavioural sleep patterns and bout structure.

## 1 Introduction

Sleep is a complex biological state involving numerous brain regions and neurotransmitters. It is regulated by a circadian process which determines sleep/wake timing, as well as a homeostatic process resulting in an increasing requirement for sleep with prolonged waking (Borbély, 1982; Borbély et al., 2016). Whilst transcriptomic studies have played a key role in understanding the genetic basis of circadian rhythms (Duffield, 2003; Hardin and Panda, 2013; Millius and Ueda, 2017), less is known about the genetic pathways involved in the regulation of sleep. Several individual transcriptomic studies comparing waking vs sleeping animals have identified sleep-state related changes in energy metabolism and stress pathways as well as a number of genes associated with synaptic plasticity (Elliott et al., 2014; Mackiewicz et al., 2009). However, a consistent pathway-level understanding is lacking.

Transcriptomics - the analysis of global patterns of gene expression - has become a key method in biological research with the increasing use of microarrays and RNA sequencing. The ability to assess transcriptional response to different stimuli has enabled the identification and characterisation of molecular mechanisms underlying physiology and behaviour. Over the past two decades, considerable research into sleep has focused on transcriptomic analysis of sleep deprived animals. Analyses of gene expression show that hundreds of transcripts are altered in sleep and waking states in species ranging from mice (Hasan et al., 2014; Terao et al., 2003; Thompson et al., 2010) and rats (Conti et al., 2006; Porter et al., 2012; Terao et al., 2006; Winrow et al., 2009), to flies (Keegan et al., 2007; Shaw et al., 2000) and humans (Möller-Levet et al., 2013). Whilst microarray studies are relatively hypothesis-free and can be considered exploratory, this approach has already resulted in the identification of key genes expressed during sleep loss, such as *Homer1* and *Cirbp* (Hoekstra et al., 2019; Maret et al., 2007). However, due to cost as well as practical considerations, individual microarray studies are often of a limited sample size, reducing statistical power to detect subtle changes. Due to differences in experimental design, as well as the bioinformatic and statistical methods used, the genes identified in individual studies frequently differ (Brown and Peirson, 2018; Duffield, 2003). As such, integrating the results of multiple microarray sleep studies is challenging. A simple way of addressing this problem is to focus on the transcripts that reproducibly change in more than one study – an approach termed ‘vote counting’ (Song and Tseng, 2014). But when multiple studies are considered, this list becomes very limited, containing just those transcripts with very large effect sizes. An alternative solution to this problem is the combination of datasets in the form of a meta-analysis (Ramasamy et al., 2008). Whilst meta-analyses are the gold standard in clinical research when different treatments are considered, they are rarely employed in basic transcriptomic biology. However, this approach has been successfully applied to identify rhythmic genes in *Drosophila* (Keegan et al., 2007) and genes enriched in the suprachiasmatic nucleus, the master circadian pacemaker in the mammalian hypothalamus (Brown et al., 2017).

Here we describe a meta-analysis of transcriptomic data from 6 published microarray studies on sleep deprivation in mice. This dataset consists of 23 pairwise comparisons of sleep vs sleep-deprived mouse samples, totalling 173 microarrays with data from 245 mice (123 sleep, 122 sleep-deprived). The outcome of this meta-analysis is a combined mean effect size and significance FDR-corrected q-values for over 28,000 genes. By combining multiple microarray datasets, we provide an integrated assessment of the effects of sleep deprivation on gene expression. Our data highlight the role of key genes known to change as a result of sleep deprivation, as well as identify new genes that have not previously been associated with sleep. We go on to validate one of these candidate genes, *Rasd1*, also known as *Dexras1*, to show that disruption of this gene in mice results in changes in the amount and distribution of behaviourally defined sleep.

## 2 Methods

### 2.1 Datasets and inclusion criteria

Microarray datasets are deposited upon publication, providing an ideal resource for transcriptomic meta-analysis. An initial search of NCBI's Gene Expression Omnibus (GEO) (Edgar et al., 2002) and the EBI's ArrayExpress (Brazma et al., 2003) identified 17 datasets on sleep transcriptomics, summarised in **Data Set S1**. Many of these datasets contained multiple experiments. Studies were then excluded that would introduce variation not due to sleep deprivation alone. For example, some datasets included rat models, stressful sleep deprivation methods as well as sleep deprivation durations up to 72 h. Only data where Minimum Information About a Microarray Experiment (MIAME) data (Brazma et al., 2001) were available were included. Inclusion criteria are summarised in Table 1. In brief, these were the presence of information about animal species (mice), strain, sex, age and number per group; sleep deprivation duration and method; brain tissue collection, time of tissue collection, microarray platform, light exposure and food availability. As age is known to affect sleep quality (Wimmer et al., 2013), the meta-analysis was limited to experiments on adult mice (one month to a year old). Beyond 12 h of sleep deprivation, changes in the light/dark cycle could affect results, increasing experimental variation. Therefore, such studies were excluded. Details of all study exclusions are reported in **Data Set S2**. After exclusion, the remaining seven public datasets from six papers yielded 173 microarrays. The datasets included in this meta-analysis are summarised in Table 2, including the relationship between datasets, microarrays, and animals**.**

**Table 1:** Meta-analysis inclusion criteria.

Feature	Inclusion criteria
Species	Mouse
Age	8-52 weeks
Duration of sleep deprivation	3-12 h
Time of day	Sleep deprivation entirely within light phase
Method of sleep deprivation	Gentle handling or novel object exposure
Time at tissue collection	Control must match sleep deprived group
Light exposure	12/12 light/dark
Food availability	*Ad libitum*

**Table 2:** **Summary of studies used for meta-analysis.** The relationship between public datasets, publications, microarrays, experimental groups, and mice alongside key experimental details. Where not otherwise stated brain tissue sampled is whole brain.

Publications	GSE	#	Experimental procedure	Total Sleep Dep	Mice (TSD:Control)	Sex	N	Microarray platform
Mackiewicz et al. (2007)	GSE6514	*1*	Cerebral cortex (TSD: ZT0-3)	3h	5:5	M	10	Affymetrix MG 430 2.0
		*2*	Cerebral cortex (TSD: ZT0-6)	6h	5:5	M	10	
		*3*	Cerebral cortex (TSD: ZT0-9)	9h	5:5	M	10	
		*4*	Cerebral cortex (TSD: ZT0-12)	12h	5:5	M	10	
		*5*	Hypothalamus (TSD: ZT0-3)	3h	5:5	M	10	
		*6*	Hypothalamus (TSD: ZT0-6)	6h	5:5	M	10	
		*7*	Hypothalamus (TSD: ZT0-9)	9h	5:5	M	10	
		*8*	Hypothalamus (TSD: ZT0-12)	12h	5:5	M	10	
Bellesi et al. (2013)	GSE48369	*9*	Forebrain excluding Oligodendrocytes	4-8h	6:6	M&F	12	Affymetrix MG 430 2.0
		*10*	Forebrain including Oligodendrocytes	4-8h	6:6	M&F	12	
Bellesi et al. (2015)	GSE69079	*11*	Astrocytes	4h	6:6	M&F	12	Affymetrix MG 430 2.0
		*12*	Non-astrocytic cells	4h	2:2	M&F	4	
Vecsey et al. (2012)	GSE33302	*13*	Hippocampus	5h	8:9	M	17	Affymetrix MG 430 2.0
Hinard et al. (2012)	GSE33491	*14*	Cerebral cortex	6h	9:9	M	6^a^	Affymetrix ME 1.0 ST
Maret et al. (2007)	GSE9441	*15*	Whole brain from AK strain mice	5.5h	9:9	M	6^a^	Affymetrix MG 430 2.0
		*16*	Whole brain from B6 strain mice	5.5h	9:9	M	6^a^	
		*17*	Whole brain from D2 strain mice	5.5h	9:9	M	6^a^	
	GSE9442	*18*	AK strain (TSD: ZT0-6)	6h	3:3	M	6^a^	Affymetrix MG 430 2.0
		*19*	B6 strain (TSD: ZT0-6)	6h	3:3	M	6^a^	
		*20*	D2 strain (TSD: ZT0-6)	6h	3:3	M	6^a^	
		*21*	AK strain (TSD: ZT6-12)	6h	3:3	M	6^a^	
		*22*	B6 strain (TSD: ZT6-12)	6h	3:3	M	6^a^	
		*23*	D2 strain (TSD: ZT6-12)	6h	3:3	M	6^a^	
**6 papers**	**7 studies**		**23 pairwise comparisons**		**122/123**		**173**	**2 platforms**

a Indicates samples from 3 mice pooled per microarray.

### 2.2 Raw data extraction and pre-processing

Raw data and key experimental details were extracted for each dataset. Raw data were extracted as ‘GSM####.CEL’ files from the GEO and ArrayExpress databases. Original microarray scan GSM###.CEL files are commonly referred to as Feature Level Extraction Output (FLEO) (Ramasamy et al., 2008). Working from raw FLEO data allows control over the processing of array files and consequently increases the accuracy of comparisons at the gene level. For Affymetrix Mouse Genome 430 2.0 (430AV2) arrays, GSM###.CEL files were imported into AltAnalyze version 2.9.0.2 and processed via integration with Affymetrix power-tools, using the Robust Multi-array Averaging (RMA-sketch) algorithm. Each of the 23 experiments was analysed individually giving the relative expression levels for both sleeping control (X_1_) and sleep-deprived (X_2_) groups for all the genes on the microarray. The output is a number of ‘DATASET#####.txt’ files, corresponding to the whole dataset. These files can contain multiple experiment sub-comparisons. For Affymetrix Mouse Exon 1.0 ST arrays, expression at the gene level was only examined for probe-sets that match to exons present in all expressed known splice variants of the ENSEMBL transcript in question. Those probe sets without mappings were excluded. Following removal of probe-sets known to cross-hybridize (x- and s-appended), the remaining probe-sets were then mapped to the ENSEMBL mouse database (build 72).

### 2.3 Meta-analysis

Transcriptomic meta-analysis was performed as described previously (Brown et al., 2017). A schematic overview of the meta-analysis workflow is shown in Fig. 1. Twenty-five original Notebooks in Jupyter Notebooks (Shen, 2014) (an IPython based web application) were written specifically to perform the meta-analysis. A benefit of working in Jupyter Notebooks is that full workings can be published online, thereby improving transparency and reproducibility of studies as well as enabling public adaptation, in the interest of open-science (Eglen et al., 2017). The analysis implements the PyData stack in Python version 3.6.7, as part of the Anaconda Python distribution version 1.9.2. Jupyter Notebooks A1 to A23 run in parallel for each of the experimental groups, performing basic calculations on the AltAnalyze output files prior to the meta-analysis. Effect sizes (ES) for each experimental group (sleep deprived vs sleeping control) are calculated as Hedges' g values (Borenstein et al., 2009). This involves calculation of Cohen's d value, followed by an adjustment factor for the number of arrays (known as the j factor). The variance of each ES is also calculated. Notebook B1 performs indexing to collate the separate Notebook “A##” outputs to a single file called ‘Symbol_forMeta.csv’. In Notebook B2, all the data are indexed against MGI gene symbols (using BioMart mouse version 72). A total of 28,220 MGI gene symbols had data for at least one microarray platform. The combined mean effect size (M∗) and significance are calculated by the inverse variance methods based on a Random Effects Model (REM) as described by Choi et al. (2003). This model does not assume that there is a single common effect size, but rather a range of true effect sizes with additional sources of variation. From the meta-analysis, Z-values were used to calculate p-values from two-tailed tests. Subsequently, the p-values were used to apply a multiple-testing correction in the form of the false discovery rate (FDR) q-value. The subsequent FDR-adjusted q-values were calculated from p-values using a q-values R package (http://qvalue.princeton.edu/).

**Fig. 1:**
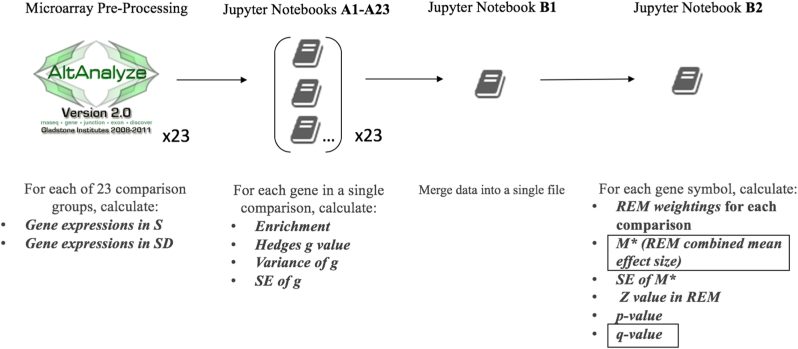
**Summary of workflow.** Pre-processed data were analysed as a series of 23 pairwise comparisons of sleep vs sleep-deprivation. For each gene an effect size (enrichment), Hedges g, variance of g and standard error of g were calculated. These results were then merged, and for each gene symbol weighted as a meta-analysis based upon a random effects model (REM). The output was a combined mean effect size (M∗) and q-value (corrected p-value).

### 2.4 Pathway analysis

Gene Ontology (GO) biological process terms and their associated genes were accessed using the GO.db package in R (Ashburner et al., 2000; Team, 2021). An over-representation analysis was conducted on the list of statistically significant genes by using Fisher's exact test with Bonferroni correction for multiple comparisons (Ashburner et al., 2000; Fisher, 1935). To correct for ascertainment bias in brain transcriptomic data, the background list of genes tested for over-representation was limited from the whole genome to include only brain enriched genes as defined by the human protein atlas (Uhlén et al., 2015). Visualization of enriched GO terms and their shared genes was performed with a gene concept network. All statistical tests and visualizations were performed in R using clusterProfiler (Yu et al., 2012).

### 2.5 Comparison with human GWAS data

The biological interpretation of GWAS loci is challenging as the single nucleotide polymorphisms (SNPs) associated with a specific trait may reflect either linkage with a nearby causative gene or regulation of gene expression via non-coding changes. As genes may be regulated by distal elements lying 10k – 100k of bases from the transcriptional start site, simply selecting the nearest gene to the genetic marker is unreliable (Tam et al., 2019). One way of addressing this challenge is to look at the enrichment of genes in genomic regions of interest at a range of genomic intervals (peak sets). This Peak set Enrichment in Gene Sets (PEGS) method (Briggs et al., 2021) can be used to compare any input list of genes against significant GWAS loci for a particular trait. As the peak set distance expands, this will include more genes, which the hypergeometric distribution corrects for (the more genes within the peak set, the greater the number of input list genes must also be present for a significant result). This approach identifies overlap between the input gene list and GWAS data across different genomic distances.

We used the PEGS approach to establish whether genes regulated by sleep deprivation could be proximal to SNPs identified in four human sleep phenotype GWAS: chronotype (https://pubmed.ncbi.nlm.nih.gov/30696823/), insomnia (Lane et al., 2019), daytime sleepiness (Wang et al., 2019), sleep duration (Lane et al., 2017). Up- and down-regulated genes were mapped to their human orthologs, and a list of these successfully mapped genes are found in **Data Set S3**. These genes were taken forward for PEGS analysis. Briefly, SNPs from six representative sleep trait GWAS with a P value below 5E-8 (commonly used in GWAS) (Xu et al., 2014) were extracted from datasets deposited in the Sleep Disorders Knowledge Portal (sleepdisordergenetics.org, 22 September 2023, RRID:SCR_016611). These SNPs were then used as the midpoint of intervals of increasing sizes that extend either side of the SNP from 5 kb to 2 Mb, based upon distances previously shown to contribute to GWAS hits (Brodie et al., 2016). The intervals’ coordinates were then intersected with the coordinates of up and down-regulated genes and the number of intersections counted. Hypergeometric test was used to calculate the significance of the intersection with total number of genes in the genome as the background. The results of this analysis are shown in Fig. 5, and a full list of P values for enrichment can be found in **Data Set S4**.

### 2.6 Sleep deprivation and tissue collection

Sixteen C57BL/6J adult male and female mice (6-8 weeks of age) were housed individually on a 12h/12h light-dark schedule with lights on at ZT0 (150 lux, cool white LED). Food and water were made available *ad libitum* for the duration of the experiment. Acclimation to experimenter presence and gentle handling was achieved by daily handling for 3-6 days before the experiment as in Vecsey et al. (2012). Handling at this point mimicked handling for sleep deprivation, and mice were not removed from their cages. The two arms of the experiment included 8 mice each with an even split of male and female mice. Sleep deprivation was carried out in the animal's home cage by gentle handling such as making mild noises or tapping or jostling the cage, disturbing nesting material or stroking the animal. To minimise direct contact, these interventions were only carried out when animals attempted to go to sleep. Non-sleep deprived mice were left undisturbed in their individual home cages. After 3 h of sleep deprivation or undisturbed sleep, animals were killed by cervical dislocation and cerebral cortex was dissected. Tissue was collected immediately following the behavioural treatment and alternated between sleep deprived and non-sleep deprived mice. Two replicate cortical samples were taken from each mouse and all tissues harvested were stored at −80°C.

### 2.7 Q-PCR validation

RNA was extracted for all 16 samples with the RNeasy Micro Kit (Qiagen) including a DNA buffering step as per the manufacturer's protocol. RNA concentration was determined by 260/280 absorbance (NanoDrop). Reverse transcription was performed following SuperScript IV VILO (ThermoFisher) protocols without DNase (which was included during RNA extraction) and cDNA samples were stored at −80°C. This cDNA was then used as a template for qPCR from which Ct and R0 were calculated as described previously (Peirson et al., 2003). TaqMan assays were used for target genes (*Arntl*, *Fastkd5*, *Hsf1*, *Mag*, *Maoa*, *Naglu*, *Rasd1* and *Tipin*), with SYBR Green qPCR for 3 housekeeping genes (*Arbp*, *Tbp* and *Gapdh*). The geometric mean of these three housekeeping genes was used for normalisation of target genes (Vandesompele et al., 2002). Triplicates for each gene were run on a single plate. The R0 mean and SEM of the triplicates were used to compare the average candidate gene expression in the sleep deprived tissue vs control tissue. Differences between control and sleep deprived samples were assessed based upon unpaired t-tests. Analysis of upregulation/downregulation was assessed using a binomial distribution.

### 2.8 *Rasd1* KO mice

All animal handling and experimental procedures were performed at the University of Toronto Mississauga Animal Facility and were approved by the Biological Sciences Local Animal Care Committee, complying with guidelines established by the University of Toronto Animal Care Committee and the Canadian Council on Animal Care. *Rasd1* knockout mice (*Rasd1* KO) were backcrossed for 13 generations onto a C57BL/6J background and thereafter maintained as a homozygous breeding colony (Cheng et al., 2004). A total of 18 *Rasd1* KO and 18 age-matched wild-type control mice on a C57BL/6J background were used for passive infrared sensor (PIR) recording, with 9 males and 9 females per genotype (Cheng et al., 2004). Mice were bred and maintained on a fixed 12-h light:12-h dark (12:12 LD) schedule in which lights on and lights off corresponded to 8 a.m. and 8 p.m. Eastern Daylight Time (EDT), respectively. The animals were maintained at 40–60% humidity and 20–24°C. Lighting conditions in animal housing rooms were 100–150 lux (cool white LED light) from floor level to workbench level. Air exchanges in the animal housing rooms and behavioural cabinets were maintained at 15–20 air changes/hour.

### 2.9 Behaviourally defined sleep scoring of *Rasd1* knockout mice

Adult mice (84 to 115 days of age) were individually housed in ventilated, light-tight cabinets under computer-controlled light schedules (Phenome Technologies), where lights on and lights off corresponded to 9 a.m. and 9 p.m. EDT, respectively. Cage-level light intensity in the cabinets was set at 140 Lux. *Rasd1* KO mice and wild-type control mice were placed in alternating order, in the same cabinet and shelf. 3 *Rasd1* KO and 3 wild-type mice of the same sex were recorded at the same time, with 36 mice recorded in total. The study was balanced, with 9 males and 9 females representing each genotype. Passive infrared (PIR) sensors were individually installed above each cage in the cabinets, recording motion every 10 s (Brown et al., 2016). Animals were given one day to acclimate to the cabinets, after which baseline recordings were taken for 7 days. On the eighth day, animals were removed from the cabinets and placed in the animal housing room for total sleep deprivation (TSD); the light intensity at cage-level was 140 lux. Animals were sleep deprived for 6 h starting at ZT0 by stroking their dorsal side or gently tapping the cage (as above). In the final 2 h of TSD, mice were transferred to a new cage with a novel object (plastic cubes). Animals were then transferred back to their recording cages and PIR recordings taken for four additional days. An animal was considered asleep if there were at least four consecutive 10-s bins of PIR inactivity. If this condition was met, the number of consecutive bins of PIR inactivity were added and converted to minutes to provide a measure of sleep duration. Sleep durations were pooled to provide a measure of how long the animal was asleep during an hourly or 6-h interval and divided by the time of the interval to calculate sleep proportion. Number of sleep bouts was defined as the number of times an animal was considered asleep in an hourly or 6-h interval. The average length of a sleep bout was defined as the amount of time an animal was asleep for during an hourly or 6-h interval divided by the number of bouts occurring within that interval. Sleep measures following sleep deprivation were calculated from 6-h bins and normalized to baseline values averaged over 7 days. Percent activity was calculated as the average of the 10-s bins of PIR readings in an hourly interval, representing the mean of 7 days of baseline activity.

### 2.10 Behavioural data analysis of *Rasd1* KO mice

Statistical analyses were conducted in R version 4.1.2. Data were combined for males and females, and Split Plot ANOVAs were conducted with genotype as the between-subject factor and ZT hour or time after sleep deprivation as the within-subject factor. The combined analysis did not include a sex-dependent effect in the model, but this was evaluated post hoc. The combined data were then filtered by sex and Split Plot ANOVAs were similarly conducted to explore potential sex-specific effects. Huynh-Feldt corrections were applied to the degrees of freedom in cases where the assumption of sphericity was violated. Post-hoc t-tests were conducted between genotypes per repeated measure and corrected using the Benjamini-Hochberg method.

## 3 Results

### 3.1 Transcriptomic meta-analysis identifies new candidate sleep genes

Our transcriptomic meta-analysis identified 498 genes whose expression significantly changed following sleep deprivation (FDR corrected q value < 0.01). These genes are visualised in Fig. 2 and all 498 significant genes are listed in **Data Set S5** and summarised in **Data Set S6**. Of these 498 genes, 219 were upregulated following sleep deprivation (M∗>0) and 279 were downregulated (M∗<0). The combined mean effect size (M∗) and FDR-adjusted q-values are shown for each gene. Ninety-six of these sleep-dependent genes were identified in the original studies included in this meta-analysis. This includes many genes that have been reproducibly shown to change in the brain as a result of sleep deprivation, including *Fos*, *Cirbp*, *Creld2*, *Crem*, *Homer1*, *Hspa5 (BiP)*, *Per2*, *Rbm3*, *Sytl2*, *Usp2* and *Xbp1*. In addition, genes identified in this meta-analysis also include genes shown in other studies to be affected by sleep deprivation, including *Arc*, *Bdnf*, *Dbp*, *Egr1*, *Egr2*, *Hspa1b*, *Nr4a1* and *Vip* (Elliott et al., 2014). Many of these genes show large amplitude changes as a result of sleep deprivation, as shown in Fig. 2. By contrast, we also identified 402 new genes that were not reported by any of the original studies used in the meta-analysis. These genes are likely to reflect lower amplitude changes that were not detected by the original studies but that are detected here due to the increased statistical power of the meta-analysis. In a typical meta-analysis, individual data points represent independent studies. It is important to note that in this transcriptomic meta-analysis we incorporated data from 6 separate papers (Bellesi et al., 2013, 2015; Hinard et al., 2012; Mackiewicz et al., 2007; Maret et al., 2007; Vecsey et al., 2012). The meta-analysis was then based upon a series of 23 pairwise comparisons of gene expression data between sleep deprived and sleeping control mice from individual matched datasets within these published papers to produce a combined mean effect size. For clarity, the results of all 23 pairwise comparisons for all 28,220 MGI gene symbols are summarised in **Data Set S7**.

**Fig. 2:**
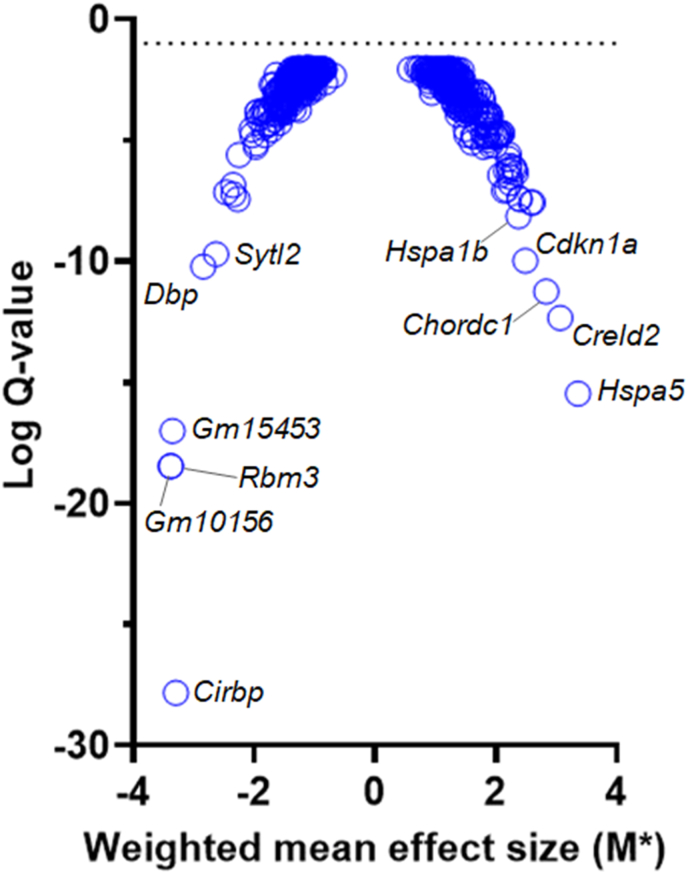
**Volcano plot of genes found to be significantly enriched and depleted in sleep deprivation by this meta-analysis.** Labels are of the top genes most significantly enriched or depleted.

### 3.2 Pathway analysis

The enrichment of GO pathways using GO.db for the 498 significant genes showed five key pathways were identified as enriched, including mRNA processing, circadian rhythm, regulation of protein catabolic process, endoplasmic reticulum unfolded protein response and negative regulation of kinase activity (Fig. 3). These pathways are consistent with a review of transcriptomic studies of sleep deprivation (Elliott et al., 2014), and include 11 genes that have been previously shown to be affected by sleep deprivation (*Bdnf*, *Cdkn1a*, *Cirbp*, *Dbp*, *Dusp1*, *Egr1*, *Hspb1*, *Hspa5*, *Per2*, *Vip* and *Xbp1*).

**Fig. 3:**
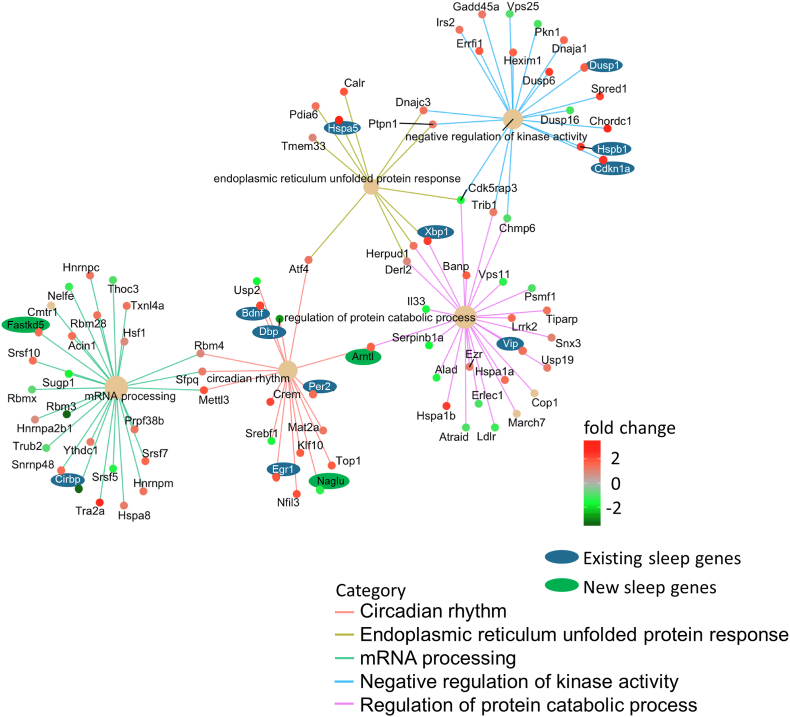
**Pathway analysis of 498 genes identified in this meta-analysis.** Enriched pathways include negative regulation of kinase activity, endoplasmic reticulum unfolded protein response, regulation of protein catabolic process, circadian rhythm and mRNA processing. Within these five pathways 11 known sleep-related genes were detected. In addition, 3 genes were identified for further analysis.

### 3.3 Identification of new candidate sleep genes

**Fig. 4:**
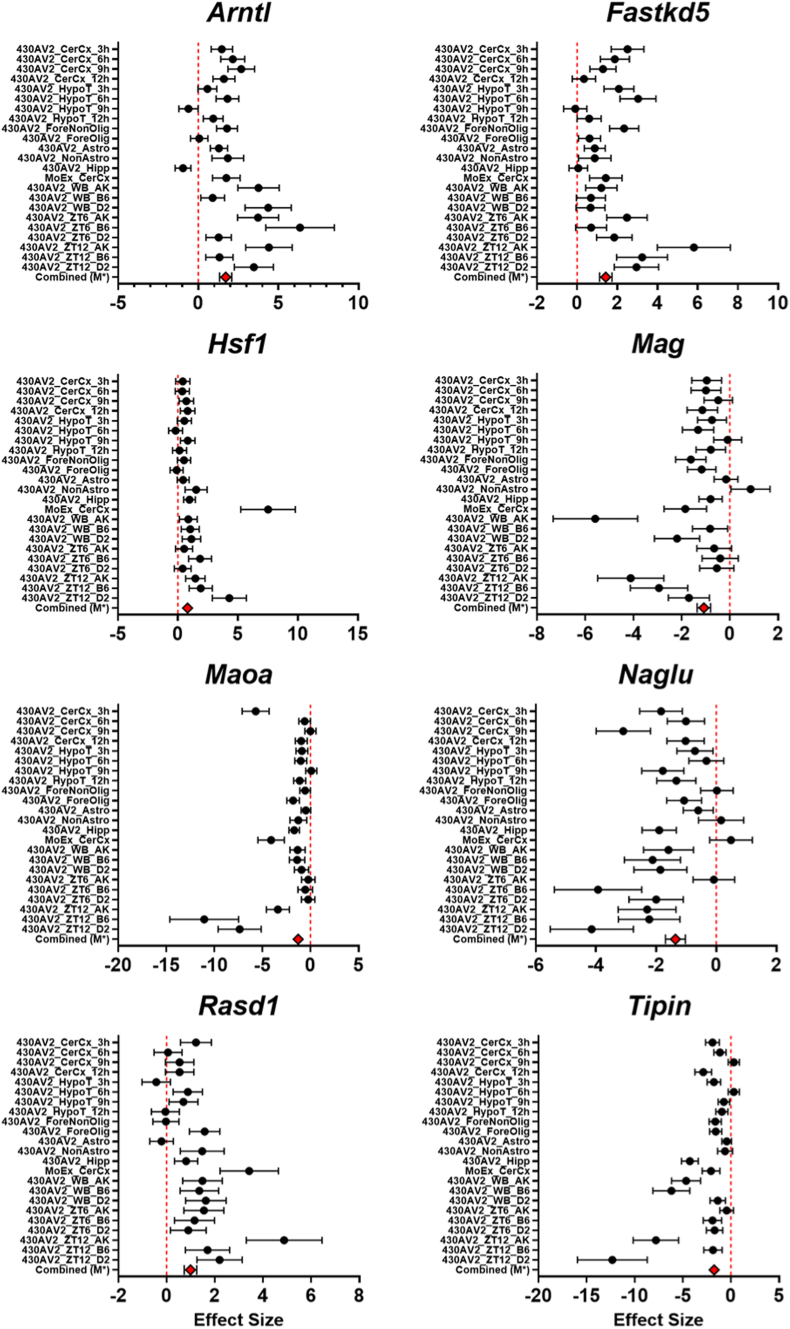
**Forest plot of the data in the meta-analysis for candidate genes of interest listed in**Table 4**.** This consists of the combined effect size (M∗, top), followed by individual Hedges' *g* values for each platform/study. Error bars show variation within each study and node size represents the weighting towards the final M∗ value.

In addition to known sleep-related genes, a number of new candidate genes were identified via pathway analysis, including *Arntl (Bmal1)*, *Fastkd5* and *Naglu*. In addition, the 402 genes uniquely identified in this study were investigated for potential mechanistic links to sleep via existing mouse genetic resources. Initially, an MGI batch query (www.informatics.jax.org/batch) of mammalian phenotypes (MP) was used to identify genes within this dataset which were associated with sleep phenotypes. Genes identified in this meta-analysis were also screened against the IMPC database (www.mousephenotype.org). Given the increasing human GWAS resources on sleep, candidate genes were also screened against the GWAS catalogue (www.ebi.ac.uk/gwas/) and the Sleep Disorders Knowledge Portal (www.sleepdisordergenetics.org/) for associations with sleep-relevant traits. This identified 14 sleep-deprivation regulated genes to be associated with sleep traits, including *Ahsa2*, *Arntl*, *Bcap29*, *Eif4b*, *Lrrk2*, *Pccb*, *Pllp*, *Rasd1*, *Rbm4*, *Srsf10*, *Tmem219*, *Ubr5*, *Zbtb16* and *Zmynd8*. These genes are summarised in Table 3. The probability of selecting 14 or more genes in this sample (498 genes) with a human association with sleep (170 out of 28,220 genes) is 2.15 x 10^−6^ (Fisher's exact test). Eight of these candidate genes were selected for further investigation based on support from both mouse and human genetic data – *Arntl*, *Fastkd5*, *Hsf1*, *Mag*, *Maoa*, *Naglu*, *Rasd1* and *Tipin* (Table 4). Forest plots for these 8 genes showing the effect sizes in each of the 23 pairwise comparisons, as well as the combined mean effect size are shown in Fig. 4. Of these candidate genes, *Arntl* and *Rasd1* were supported by human GWAS associations.

**Table 3:** Genes identified in this study with human genetic associations with sleep traits.

Gene	Gene Name	M∗	Q-value	Genetic associations	PMID
*Ahsa2*	AHA1, activator of heat shock protein ATPase 2	*2.21*	*2.57E-06*	Associated with "Morning person" trait	30696823
*Arntl*	*Aryl hydrocarbon receptor nuclear translocator-like*	*1.71*	*0.00090*	*Associated with "Chronotype" trait* *Associated with diabetes mellitus*	30696823
*Bcap29*	B cell receptor associated protein 29	*−1.15*	*0.0082*	Associated with "Sleep duration" trait	30804565
*Eif4b*	Eukaryotic translation initiation factor 4B	*−1.23*	*0.0030*	Associated with "Night sleep phenotypes" trait	27126917
*Lrrk2*	*Leucine-rich repeat kinase 2*	*1.49*	*0.00020*	*Associated with "Daytime sleep phenotypes" trait*	27126917
*Pccb*	Propionyl Coenzyme A carboxylase, beta polypeptide	*−1.3*	*0.0044*	Associated with "Sleep duration" trait. Also associated with Parkinson's.	30804565 26965688
*Pllp*	Plasma membrane proteolipid	*−2.35*	*5.75E-08*	Associated with "Sleep duration" trait	23728906
*Rasd1*	*RAS, dexamethasone-induced 1*	*0.99*	*0.0082*	*Associated with "Chronotype" trait* *Associated with "Morning person" trait* *Associated with "Morningness" trait* *Association with Parkinson's disease*	30696823 30696823 30804565
*Rbm4*	RNA binding motif protein 4	*0.92*	*0.0056*	Associated with "Insomnia" trait RBM4B is associated with "Insomnia" trait	30804565 30804565
*Srsf10*	Serine and arginine-rich splicing factor 10	*1.48*	*0.00093*	Associated with "Chronotype" trait	30696823
*Tmem219*	*Transmembrane protein 219*	*−1.13*	*0.0072*	Associated with "Insomnia" trait	30804565
*Ubr5*	Ubiquitin protein ligase E3 component n-recognin 5	*−1.18*	*0.0082*	Associated with "Insomnia" trait	30804565
*Zbtb16*	*Zinc finger and BTB domain containing 16*	*1.85*	*1.39E-05*	Associated with "Chronotype" trait Associated with "Morning person" trait Associated with "Morningness" trait	30696823 30595370 30804565

**Table 4:** **Novel candidate sleep genes identified in this study.** The effect size of the change and evidence for potential links to sleep are also summarised.

MGI Symbol	Gene Name	M∗	Q-value	Evidence	PMID
*Arntl*	*Aryl hydrocarbon receptor nuclear translocator-like*	*1.71*	*0.00090*	Final clock gene to be associated with sleep deprivation. *Abnormal sleep pattern; fragmentation of sleep/wake states; abnormal circadian regulation of heart rate; abnormal circadian regulation of systemic arterial blood pressure; abnormal circadian behaviour; shortened circadian behaviour period; arrhythmic circadian behaviour persistence; abnormal circadian behaviour phase;* *delayed circadian behaviour phase;* *abnormal locomotor circadian rhythm;* *abnormal circadian sleep/wake cycle.*	9616112 16171284 30696823
*Fastkd5*	*FAST kinase domains 5*	*1.43*	*3.69E-06*	Abnormal sleep (JAX lab) IMPC data release 10.0	27626380
*Hsf1*	*Heat shock factor 1*	*0.81*	*0.0084*	Abnormal sleep (JAX lab) IMPC data release 10.0	27626380
*Mag*	*Myelin-associated glycoprotein*	*−1.08*	*0.0053*	Abnormal sleep (JAX lab) IMPC data release 10.0	27626380
*Maoa*	*Monoamine oxidase A*	*−1.28*	*0.0017*	*Abnormal sleep pattern*	*7792602*
*Naglu*	*Alpha-N-acetylglucosaminidase*	*−1.36*	*0.0035*	*Abnormal suprachiasmatic nucleus morphology; abnormal circadian behaviour; delayed circadian behaviour phase*	17712420
*Rasd1*	*RAS, dexamethasone-induced 1*	*0.99*	*0.0082*	*Shortened circadian behaviour period; advanced circadian behaviour phase; abnormal circadian behaviour entrainment.* Also known as Dexras1 which Cheng et al. (2004) states Dexras1 "modulates the responses of the master clock to the circadian timekeeping system."	15339652 15339652
*Tipin*	*Timeless interacting protein*	*−1.75*	*0.00017*	Interaction with Tim (abnormal sleep and circadian behaviour in mouse and human)	1138685

M∗ Effect size.

IMPC data can be queried by phenotype or by genes at: https://www.mousephenotype.org.

**Fig. 5:**
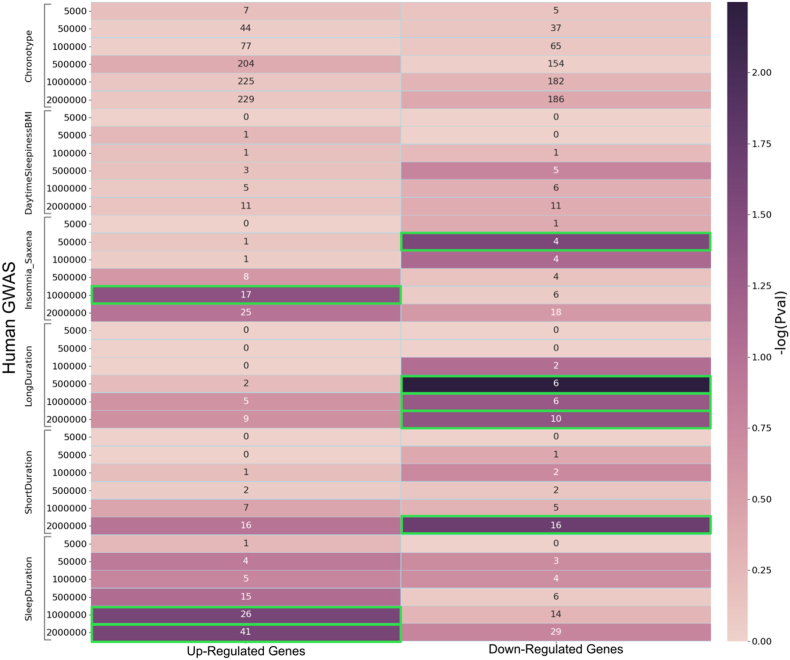
**PEGS analysis showing the number of both up- and down-regulated genes intersecting with intervals around SNPs from six sleep GWAS.** GWAS phenotype and interval are indicated on the left, the number of genes intersecting the intervals are shown in the boxes, and the significance of enrichment is coloured by -log (P value) as indicated by the scale on the right. Significant (P < 0.05) intervals are indicated by a green box and gene lists for these intervals are provided in Data Set S8.

### 3.4 Measures of heterogeneity

The combined mean effect (M∗) is reported as a summary measure in file **Data Set S6**. The effect size of each individual study is reported as Hedges g, which is summarised in **Data Set S5** (Hedges g sheet). Here the individual difference in each of the 23 pairwise comparisons is summarised for every gene. This data is illustrated in the Forest plots for the 8 candidate genes of interest in Fig. 4. As can be seen from this sample, M∗ provides an overall summary, but does not capture the heterogeneity across all studies. The inverse variance weighting model used for the meta-analysis takes the variance in each pairwise comparison into account when the M∗ is calculated, but this does not capture the variance within the 23 individual pairwise comparisons. To assess the heterogeneity of effects across the studies making up the meta-analysis, we determined the direction of change (upregulated vs downregulated) across all 23 pairwise comparisons for each gene. This identified a high degree of consistency across the 23 pairwise comparisons, with all genes showing consistent changes in at least 19/23 pairwise comparisons, and the majority of genes (86%) showing consistent changes in at least 21/23 pairwise comparisons. To also investigate the magnitude of these changes, we also calculated the standard deviation as a measure of variance of the Hedges g statistic across studies. We used this measure of variance to rank genes from 1 (lowest variance across studies) to 498 (greatest variance). These findings are summarised in Table S1. We could identify no consistent relationship between measures of heterogeneity and specific pairwise comparisons or studies.

### 3.5 Comparison with human GWAS data

There was significant enrichment (P < 0.05, indicated by green-boxed numbers in Fig. 5) in eight intervals for SNPs associated with insomnia, long sleep duration, short sleep duration, and sleep duration. Gene lists for these 8 significant enrichments are found in **Data Set S8**. Enrichment may indicate that these sleep phenotype SNPs impact genes identified in the sleep meta-analysis, either through alterations to their coding regions, or by altering cis-regulatory elements such as promoters or enhancers that regulate their expression. Gene Ontology analysis (Ashburner et al., 2000) was performed using PANTHER Overrepresentation Test (Released, 2024-01-17) (Thomas et al., 2022) on these eight gene lists relative to all *Homo sapiens* genes in the PANTHER database using a Fisher's Exact test with False Discovery Rate (FDR) correction. Only the gene list from the Short Duration SNPs with an interval of 2 Mb showed significant pathway enrichment for processes within the GO biological process (complete) dataset such as negative regulation of apoptosis, unfolded protein response, and response to endoplasmic reticulum stress (consistent with previous analysis, see Fig. 3). Full GO enrichment results for this enrichment can be found in **Data Set S9**.

### 3.6 Validation of new candidate sleep genes

**Fig. 6:**
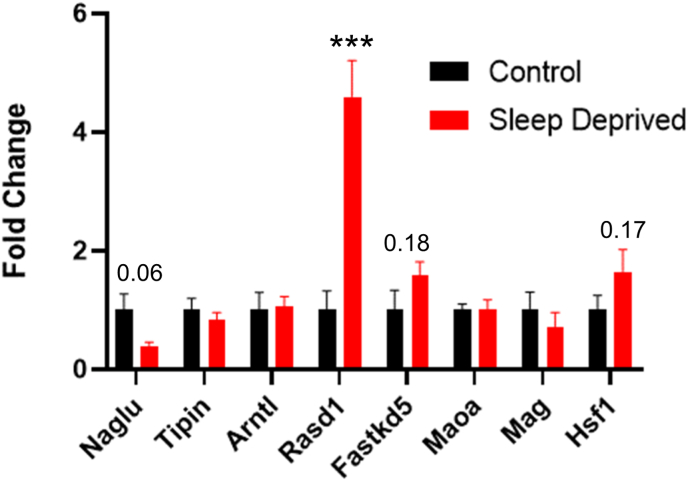
**Q-PCR validation of the 8 candidate sleep genes listed shown in**Fig. 4**and**Table 4**(n=8/group).** ∗∗∗ = P < 0.001.

To validate our findings, we conducted a 3h sleep deprivation study on adult mice (8 sleep deprived vs 8 sleeping controls) and quantified the expression of the 8 new candidate sleep genes in the cerebral cortex using qPCR (Fig. 6). Sleep deprivation is only possible during the light phase when sleep pressure is high, so the only variable affecting time of day would be the length of sleep deprivation. The majority of studies used 3-6h of sleep deprivation from ZT0 (collected ZT3-6), and as the qPCR validation tissue was collected at ZT3, this is not expected to show circadian differences in expression of clock controlled genes. A small study using 16 mice lacks power to discover genes, compared to a meta-analysis based on 245 animals, and does not capture the range of different brain regions used in the meta-analysis. However, this approach does permit limited target validation. Here, we were able to detect significant changes in one of these 8 candidate sleep genes, *Rasd1*, which showed a 4.6 fold increase in response to sleep deprivation by qPCR (meta-analysis M∗ = 0.99 on log2 scale). However, the direction of the changes observed across the 8 genes were conserved in six of the remaining seven genes (Binomial test P = 0.035), with only *Maoa* showing a change in orientation of response, with a slight increase in expression (1.02 fold) with qPCR vs a decrease in expression in the meta-analysis (meta-analysis M∗ −1.30). Based on unpaired Student's t-tests (df = 14), the effect size and p-value were as follows: Naglu (t = 2.0778, p = 0.0566); Tipin (t = 0.7465, df = 14, p = 0.4677); Arntl (t = 0.1931, p = 0.8497); Rasd1 (t = 4.7441, p = 0.0003); Fastkd5 (t = 1.3970, p = 0.1842); Maoa (t = 0.0960, p = 0.9249); Mag (t = 0.7485, p = 0.4665) and Hsf1 (t = 1.4477, df = 14, p = 0.1697). The mice used for this qPCR analysis included both male and female animals, though no sex differences were evident in the qPCR data.

### 3.7 Behavioural analysis of *Rasd1* KO mice

**Fig. 7:**
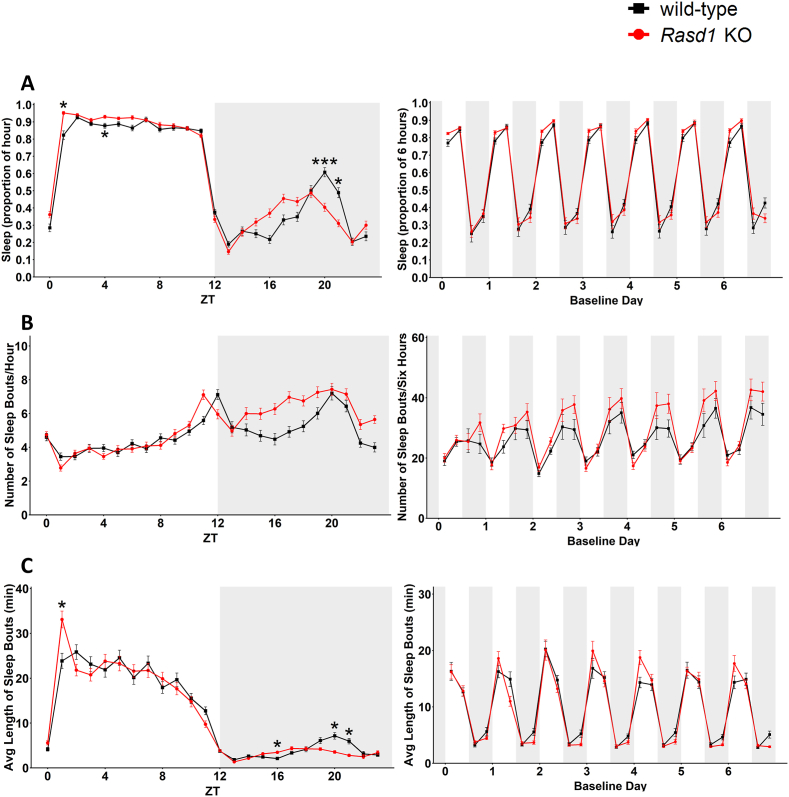
**Baseline behavioural sleep phenotyping in *Rasd1* KO mice.** Data pooled into 1-h bins averaged over 7 sequential days of 12:12 LD (left) or 6-h bins over 7 days (right). Data are combined for both sexes, with 9 males and 9 females of each genotype, representing 18 wild-type and 18 *Rasd1* KO mice. (**A**) Average proportion of behaviourally defined sleep, (**B**) average number of bouts, and (**C**) average length of sleep bouts in minutes. Values represent mean ± SEM. Statistical analyses for all behavioural measures are provided in Data Set S10. ∗∗∗p ≤ 0.001; ∗∗p ≤ 0.01; ∗p < 0.05.

The convergence of mouse and human data supporting *Rasd1* (also known as *Dexras1*) as a candidate sleep gene prompted us to investigate the consequence of *Rasd1* ablation on sleep patterns, before and after sleep deprivation. Behaviourally-defined sleep scoring was achieved using PIR sensors, as described previously (Brown et al., 2016). Baseline behavioural sleep patterns were significantly, albeit modestly, altered by *Rasd1* ablation (Fig. 7A, left, **Data Set S10**). Compared to age-matched wild-type controls, *Rasd1* KO mice slept more during the early day, at ZT 1 and ZT 4, and less during the late night, at ZT 20 and 21 (Fig. 7A, left). *Rasd1* KO mice also tended to have more night-time sleep bouts, although this effect was not significant (Fig. 7B, left). Sleep bout length was significantly longer in *Rasd1* KO mice at ZT 1, and shorter at ZT 20 and ZT 21, accounting for the differences in sleep duration between mutant and control animals at these time points (Fig. 7C, left). Separate analyses on male and female mice revealed that the aforementioned differences in behaviourally defined sleep patterns were mainly driven by changes in *Rasd1* KO females rather than males (Fig. S1A–F). Importantly, these differences in baseline sleep patterns were consistently observed across 7 days of PIR recording, indicating that *Rasd1* is required for maintaining normal sleep patterns under baseline conditions, particularly in females (Fig. 7A–C, right, and Fig. S1A–F). These differences in baseline sleep patterns are coexistent with altered activity patterns as detected by the PIRs, also driven largely by females (Fig. S3A–C).

**Fig. 8:**
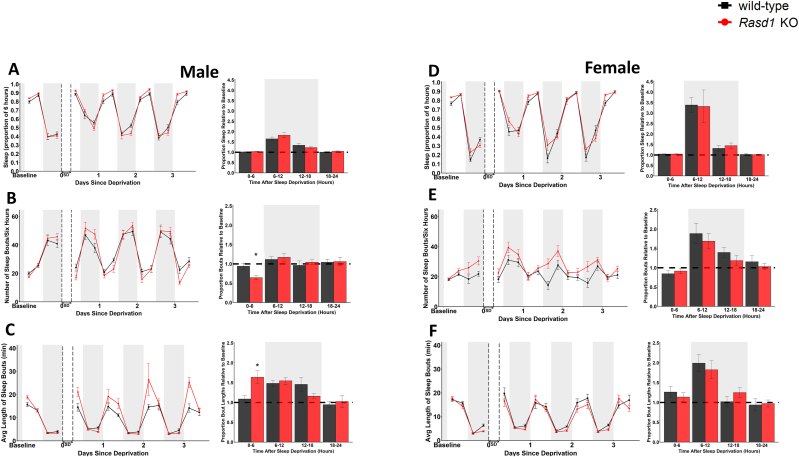
**Effects of sleep deprivation in *Rasd1* KO mice.** Data are pooled into 6-h bins. Baseline points are averaged over 7 sequential days of 12:12 LD (left). Animals were sleep deprived for 6 h beginning from ZT0 and recorded for four subsequent days (left). Data are shown separately for 9 males (**A-C**) and 9 females (**D-F**) in each genotype. Bar graphs (right) show the immediate hours following sleep deprivation, relative to baseline. (**A,D**) Average proportion of behaviourally defined sleep, (**B,E**) average number of bouts, and (**C,F**) average length of sleep bouts in minutes. Values represent mean ± SEM. Statistical analyses for all behavioural measures are provided in Data Set S10. ∗p < 0.05.

We next determined the role of *Rasd1* in sleep deprivation-induced sleep rebound following 6 h of TSD from ZT 0 to ZT 6. There was no marked difference in sleep rebound and sleep patterns between *Rasd1* KO mice and wild-type controls in the 72h following TSD when sex was not considered (Fig. S2, Data Set S10). However, we again noted a sex-specific effect of *Rasd1* deficiency on TSD-induced sleep patterns (Fig. 8). Although sleep duration remained comparable between genotypes when sex was considered, *Rasd1* KO males exhibited a significant reduction in number of sleep bouts along with a significant increase in sleep bout length in the 6 h immediately following TSD (Fig. 8A–C, right). We interpret these results to mean that, while sleep duration was not affected, *Rasd1* KO males had more uninterrupted sleep following TSD compared with wild-types. On the other hand, *Rasd1* KO females were indistinguishable from wild-type controls in all measures of sleep rebound (Fig. 8D–F, right). Collectively, our observations reveal that *Rasd1* is critical for regulating TSD-induced sleep rebound in males, whereas in females it affects baseline sleep patterns.

## 4 Discussion

Here we describe a meta-analysis of transcriptomic data from published studies on sleep deprivation in the mouse brain. The advent of well-curated, publicly available gene expression datasets permits very well-powered gene expression analysis. Our current study consists of 23 pairwise comparisons of sleep vs sleep-deprived mouse datasets, totalling 173 microarrays with data from 245 mice, providing the largest analysis to date of the effects of sleep deprivation on gene expression in the mouse. We identified 498 differentially expressed genes in sleep deprived samples, including many well-characterised sleep-relevant genes. This included immediate early genes (*Arc*, *Egr1*, *Egr2*, *Fos* and *Nr4a1/Nurr77*), genes related to cellular stress and the unfolded protein response (*Cirbp*, *Hspa1b*, *Hspa5/BiP*, *Xbp1*), circadian clock-related genes (*Crem*, *Dbp*, *Per2* and *Vip*), plasticity-related genes (*Arc*, *Bdnf, Egr1, Homer1*) and hormone-related genes (*Dio2*, *Sgk1*)*.* Critically, we identified a large number of genes that had not been previously shown to change as a result of sleep deprivation.

Over the last five years, a large number of genome wide association studies (GWAS) have been published for sleep-related traits (Dashti et al., 2019; Doherty et al., 2018; Jones et al., 2016, 2019; Lane et al., 2017, 2019; Wang et al., 2019), providing online resources for sleep genetics such as the Sleep Disorders Knowledge Portal (www.sleepdisordergenetics.org/). These studies have identified more than 100 human genomic loci associated with sleep traits, and efforts to map the causative variant, and find the effector genes have focussed on human statistical genetics. However, independent studies in a mammalian model of sleep may identify candidate sleep genes which can be used to inform the human sleep gene identification. In our current study we find 14 genes with evidence of human genetic associations with sleep-related traits. These data provide an important link between the genetic associations and underlying biological mechanisms regulating sleep. Based on these human genetics data as well as evidence from mouse phenotyping projects and known signalling pathways associated with sleep, 8 genes were selected for further investigation. This included *Arntl*, *Fastkd5*, *Hsf1*, *Mag*, *Maoa*, *Naglu*, *Rasd1* and *Tipin*. Of these candidates, *Rasd1* was found to be highly upregulated by sleep deprivation. One reason for the elevation of *Rasd1* in response to sleep deprivation may be related to stress responses to the sleep deprivation protocol. The use of gentle handling has been shown to elevate corticosterone in mice (Longordo et al., 2011) and *Rasd1* is rapidly induced following glucocorticoid stimulation (Kemppainen and Behrend, 1998).

*Rasd1* is one of the genes affected in Smith-Magenis syndrome, a microdeletion on chromosome 17. Whilst Smith-Magenis syndrome affects a number of genes, including disruption of CLOCK transcription via RAI1 (Williams et al., 2012), this condition is characterised by intellectual disability and sleep disorders, and inverted melatonin profiles have also been described (De Leersnyder et al., 2001). Moreover, *Rasd1* knockout mice show defects in circadian rhythms, with a shorter circadian period and aberrant responses to photic cues thought to be due to impaired or altered NMDA and PACAP receptor signalling (Cheng et al., 2004, 2006). These animals also show enhanced responses to non-photic cues via NPY (Cheng et al., 2004). To date, sleep has never been examined in *Rasd1*-deficient mice. Here we show that mice lacking *Dexras1* show a subtle but reproducible sleep phenotype, characterised by changes in the amount and distribution of behaviourally defined sleep duration and sleep bout structure.

Our data suggest that *Rasd1* may promote wakefulness at times of high sleep pressure. In *Rasd1* KO females the effect is more pronounced under baseline conditions, whereas in *Rasd1* KO males the effect is observed following sleep deprivation and results in more uninterrupted behavioural sleep. Importantly, such differences in baseline behavioural sleep recordings in *Rasd1* KO females is also in conjunction with altered behavioural activity patterns. However, the exact molecular mechanisms for this are unclear. *Rasd1* has been shown to affect heterotrimeric G protein signalling, activating heterotrimeric G_i_ proteins in the absence of G protein coupled receptor (GPCR) activation (Cismowski et al., 1999, 2000) and, conversely, inhibiting ligand-stimulated GPCR-G_i_ signalling (Takesono et al., 2002). The latter effect may result in enhanced GPCR-G_i_ signalling in *Rasd1* KO mice, as demonstrated in prior studies of these animals (Cheng et al., 2004, 2006). Given the role of G_i_ coupled inhibitory A_1_ adenosine receptors in the regulation of both circadian rhythms and sleep (Jagannath et al., 2021), this may provide a potential pathway by which *Rasd1* modulates sleep. *Rasd1* may also impact other signalling pathways, including adenylyl cyclase and consequently GPCR-mediated G_s_ signalling (Cheng et al., 2006; Graham et al., 2004; Harrison and He, 2011), ERK1/2 (Cheng et al., 2004, 2006; Graham et al., 2002) and nNOS (Fang et al., 2000), all of which can modulate sleep (Cespuglio et al., 2012; Jagannath et al., 2021; Mikhail et al., 2017). Future studies are clearly required to identify the detailed pathways by which *Rasd1* regulates both sleep and circadian rhythms. However, *Rasd1* may act as a signal integrator, responding to different environmental stimuli to modulate sleep responses.

We previously found that the use of passive-infrared sensors correlates well with EEG-defined sleep (Pearson correlation >0.95) (Brown et al., 2016). Although EEG-defined sleep is important for any detailed sleep analysis, there is a growing interest in finding affordable, non-invasive measures of sleep to enable high-throughput sleep phenotyping. In this study, PIR sensors were used to monitor *Rasd1*-deficient mice, with an advantage of being able to record for extended periods of time. One of the limitations of this study is that PIRs do not measure sleep architecture, NREM vs REM sleep, spectral changes, slow wave activity associated with sleep pressure or quiet wakefulness.

Whilst the meta-analysis presented here provides a global overview of the effects of sleep deprivation on the brain, it is clearly comprised of a series of independent comparisons involving different brain regions or cell types, while also being restricted in the majority of cases to male mice (Table 2). Limitations of this approach are that it may over-represent one tissue type, such as cerebral cortex. Alternatively, the use of different starting material may introduce additional sources of variation, and real differences with small effect sizes may be overlooked. This limitation is in part unavoidable due to the studies available for such a meta-analysis. The use of a random effects model adjusted weightings may partially mitigate these issues, as this anticipates a range of true effect sizes with additional sources of variation (Borenstein et al., 2009). We found no consistent relationship between measures of heterogeneity and specific pairwise comparisons suggesting that no individual study was skewing the results. However, we found that many of the sleep regulated genes previously identified (Elliott et al., 2014) showed high combined effect sizes (M∗) but considerable heterogeneity across the 23 pairwise comparisons (SD Hedges g). By contrast, the 8 candidate genes of interest identified highlighted in our study showed lower combined effect sizes but less heterogeneity across the 23 pairwise comparisons. These findings may be expected from a meta-analysis, which combines multiple datasets to improve statistical power, detecting small consistent changes that individual more variable studies may miss (Brown and Peirson, 2018).

Moreover, not all transcripts are represented on any one microarray platform, and differences may also exist between the specificity and accuracy of specific probe sets. It is anticipated that as additional datasets become available, meta-analyses of sleep transcriptomic data may improve in resolution as well as enable analysis of subsets of data – for example, in specific brain regions or cell types, or following different durations of sleep deprivation. As well as the technical limitations described above, there are also biological limitations of a transcriptomic analysis of sleep, which obviously ignores potential translational and post-translational mechanisms. Recently published data at the level of the forebrain synapse have highlighted key differences between transcriptome and proteome, as well as the phosphoproteome (Brüning et al., 2019; Noya et al., 2019). In addition, recent efforts to understand the molecular pathways regulating sleep have applied systems genetics approaches, combining genomic, transcriptomic, metabolomics and phenomic datasets (Jan et al., 2019). Such systems approaches may improve the integration between the animal model and human genetic datasets.

Together, the targets described above provide new candidate sleep genes which have not previously been linked with sleep deprivation. Two of these genes – ARNTL and RASD1 – have been associated with human sleep-related traits. In addition, *Maoa*, *Naglu*, *Rasd1* and *Tipin* have all been linked with human disorders that show sleep disturbances. At the level of animal models, detailed sleep studies have only been conducted in *Bmal1* knockout mice, though preliminary IMPC sleep data for *Fastkd5*, *Hsf1* and *Mag* all suggest abnormal sleep physiology. Finally, *Arntl*, *Hsf1*, *Naglu*, *Rasd1* and *Tipin* have all been linked with circadian phenotypes in mouse models. These findings emphasise the interactions between the circadian and homeostatic processes in sleep regulation (Borbély et al., 2016), and, more importantly, suggest that circadian physiology may influence responses to sleep deprivation. The meta-analysis of microarrays presented here provides the largest individual analysis of sleep deprivation transcriptomic data to date, totalling 173 microarrays with data from 245 mice. Here we have identified 498 genes that are significantly affected by sleep deprivation, including 402 genes that have previously not been linked to sleep deprivation. These new candidate sleep genes include 14 genes with human genetic associations with sleep-dependent traits, as well as 3 genes for which emerging IMPC data indicate a sleep phenotype in knockout mice. The genes identified in this study provide *bona fide* candidates for future sleep studies, as well as for detailed follow-up sleep analysis using EEG/EMG. Our findings may provide a valuable resource for future research on the molecular mechanisms underlying sleep as well as the consequences of sleep deprivation. To facilitate this, we have included an additional supplementary data user guide, summarising all the data sets and providing examples as to how researchers could use this data as a resource.

## Ethics approval statement

All animal handling and experimental procedures pertaining to *Rasd1* KO mice were performed at the University of Toronto Mississauga Animal Facility and were approved by the Biological Animal Care Committee, complying with guidelines established by the University of Toronto Animal Care Committee and the Canadian Council on Animal Care. The sleep deprivation tissue collection was conducted at the University of Oxford and was approved by the local Animal Welfare and Ethical Review Body (AWERB). This work was conducted in accordance with the UK Animals (Scientific Procedures) Act under licences PP0911346 and I869292DB and in accordance with the University of Oxford Policy on the Use of Animals in Scientific research.

## Funding statement

SNP was funded by Wellcome (106174/Z/14/Z and 227093/Z/23/Z) and a Skills and Knowledge Transfer Grant from the National Centre for Replacement, Refinement and Reduction of Animals in Research (NC3Rs grant NC/V000977/1). SNP and DWR were supported by grants from the Biotechnology and Biological Sciences Research Committee (BBSRC grants BB/S015817/1 & BB/X002357/1). HYMC received grants from the Canadian Institutes of Health Research (PJT-166046 & PJT-183946) and the Natural Sciences and Engineering Research Council of Canada (RGPIN-2016-05563 and RGPIN-2024-04487). DWR is funded/supported by the National Institute for Health and Care Research (NIHR) Oxford Health Biomedical Research Centre. The views expressed are those of the author(s) and not necessarily those of the NIHR or the Department of Health and Social Care. NIHR Oxford Health Biomedical Research Centre grant reference number: NIHR203316**.** The funders had no role in study design, data collection and analysis, decision to publish, or preparation of the manuscript.

## CRediT authorship contribution statement

**Osama H.M.H. Abdalla:** Investigation, Visualization, Writing – original draft, Writing – review & editing. **Ella Dunlop:** Investigation, Methodology, Visualization, Writing – original draft. **Tatiana S. Wilson:** Investigation, Visualization, Writing – original draft, Writing – review & editing. **Paul K. Reardon:** Investigation, Methodology, Visualization, Writing – review & editing. **Mudassar Iqbal:** Investigation, Methodology, Visualization, Writing – review & editing. **Shu K.E. Tam:** Investigation, Writing – review & editing. **Vladyslav V. Vyazovskiy:** Supervision, Writing – review & editing. **David W. Ray:** Supervision, Writing – review & editing. **Laurence A. Brown:** Conceptualization, Methodology, Supervision, Writing – review & editing. **Hai-Ying Mary Cheng:** Supervision, Writing – original draft, Writing – review & editing. **Stuart N. Peirson:** Conceptualization, Supervision, Visualization, Writing – original draft, Writing – review & editing.

## Declaration of competing interest

The author is an Editorial Board Member/Editor-in-Chief/Associate Editor/Guest Editor for this journal and was not involved in the editorial review or the decision to publish this article.

The authors declare the following financial interests/personal relationships which may be considered as potential competing interests: SNP has received consulting fees from NASA Ames and Sleep Standards. All other authors declare they have no conflicts of interest.

## Figures and Tables

**figs1:**
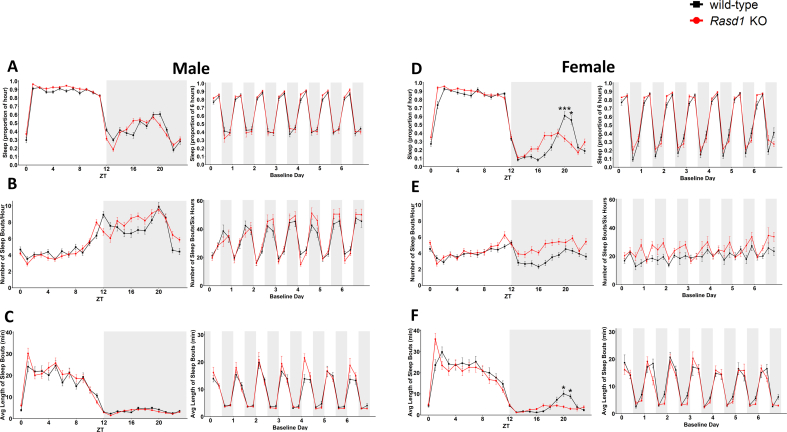


**figs2:**
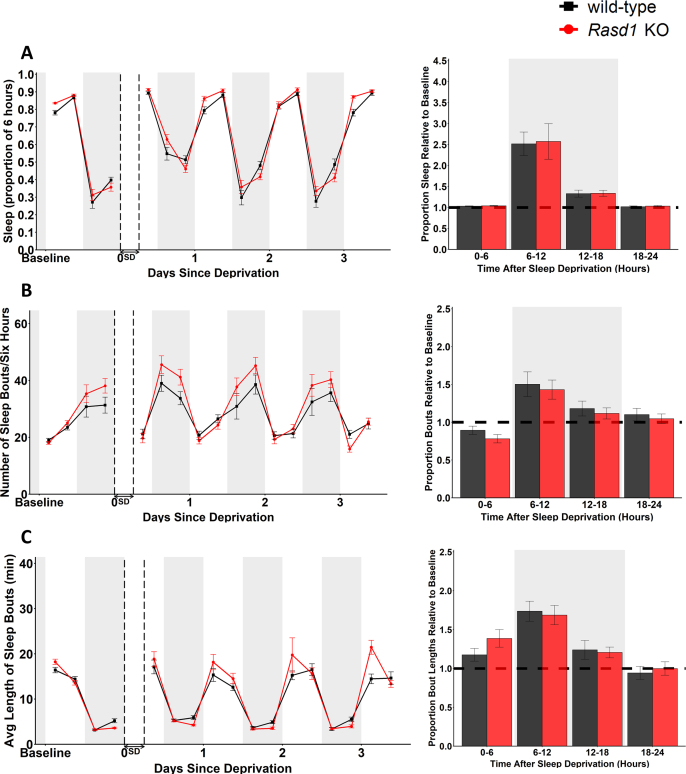


**figs3:**
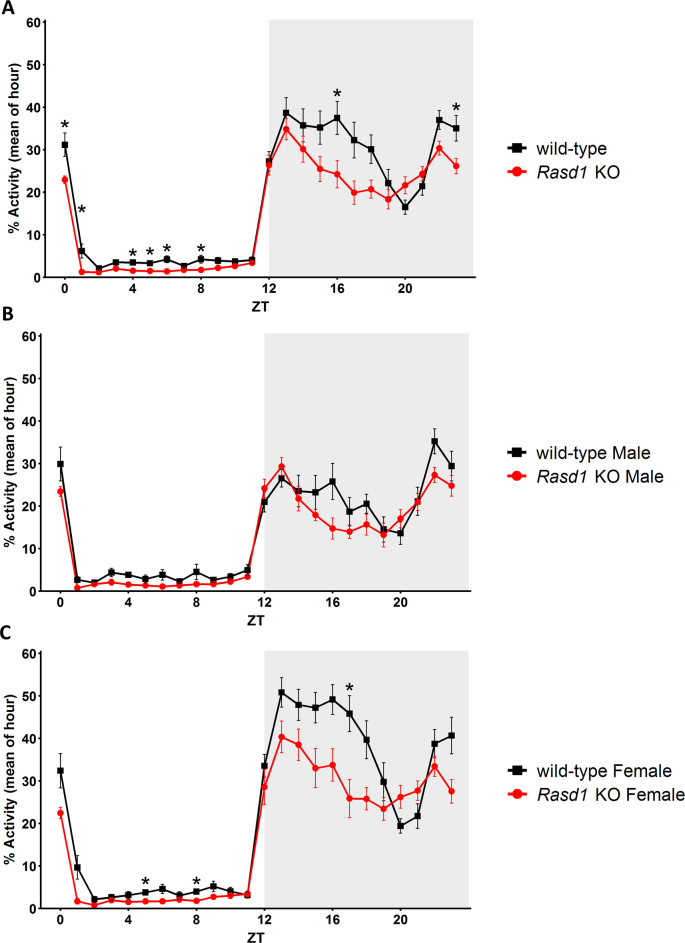


## Data Availability

All code, including Jupyter Notebooks and data files and their corresponding Conda Environments are available for download and re-production at https://github.com/FHSProject139/FSH-Project-Notebooks-and-Files, and a version of the code at the point of manuscript submission is available at https://figshare.com/s/7bed11236e44b6995564. Jupyter Notebooks and data files pertaining to the *Rasd1* KO mice can be found at https://github.com/ohabdalla1/Dexras1_Behavioural_Sleep and a version of the code at the point of manuscript submission is available at https://figshare.com/s/d5cadb1b22ff74f90265. Data used for the meta-analysis are publicly available and accession codes are listed in Data Set S1 and Data Set S2. All other data are available in the main text or the supplementary materials.

## References

[bib1] Ashburner M., Ball C.A., Blake J.A., Botstein D., Butler H., Cherry J.M., Davis A.P., Dolinski K., Dwight S.S., Eppig J.T., Harris M.A., Hill D.P., Issel-Tarver L., Kasarskis A., Lewis S., Matese J.C., Richardson J.E., Ringwald M., Rubin G.M., Sherlock G. (2000). Gene ontology: tool for the unification of biology. The gene ontology consortium. Nat. Genet..

[bib2] Bellesi M., de Vivo L., Tononi G., Cirelli C. (2015). Effects of sleep and wake on astrocytes: clues from molecular and ultrastructural studies. BMC Biol..

[bib3] Bellesi M., Pfister-Genskow M., Maret S., Keles S., Tononi G., Cirelli C. (2013). Effects of sleep and wake on oligodendrocytes and their precursors. J. Neurosci..

[bib4] Borbély A.A. (1982). A two process model of sleep regulation. Hum. Neurobiol..

[bib5] Borbély A.A., Daan S., Wirz‐Justice A., Deboer T. (2016). The two‐process model of sleep regulation: a reappraisal. J. Sleep Res..

[bib6] Borenstein M., Hedges L.V., Higgins J.P.T., Rothstein H.R. (2009).

[bib7] Brazma A., Hingamp P., Quackenbush J., Sherlock G., Spellman P., Stoeckert C., Aach J., Ansorge W., Ball C.A., Causton H.C., Gaasterland T., Glenisson P., Holstege F.C.P., Kim I.F., Markowitz V., Matese J.C., Parkinson H., Robinson A., Sarkans U., Schulze-Kremer S., Stewart J., Taylor R., Vilo J., Vingron M. (2001). Minimum information about a microarray experiment (MIAME)—Toward standards for microarray data. Nat. Genet..

[bib8] Brazma A., Parkinson H., Sarkans U., Shojatalab M., Vilo J., Abeygunawardena N., Holloway E., Kapushesky M., Kemmeren P., Lara G.G., Oezcimen A., Rocca-Serra P., Sansone S.-A. (2003). ArrayExpress--a public repository for microarray gene expression data at the EBI. Nucleic Acids Res..

[bib9] Briggs P., Hunter A.L., Yang S.-H., Sharrocks A.D., Iqbal M. (2021). PEGS: an efficient tool for gene set enrichment within defined sets of genomic intervals. F1000Research.

[bib14] Brüning F., Noya S.B., Bange T., Koutsouli S., Rudolph J.D., Tyagarajan S.K., Cox J., Mann M., Brown S.A., Robles M.S. (2019). Sleep-wake cycles drive daily dynamics of synaptic phosphorylation. Science.

[bib10] Brodie A., Azaria J.R., Ofran Y. (2016). How far from the SNP may the causative genes be?. Nucleic Acids Res..

[bib11] Brown L.A., Hasan S., Foster R.G., Peirson S.N. (2016). COMPASS: Continuous open mouse phenotyping of activity and sleep status. Wellcome Open Res..

[bib13] Brown L.A., Williams J., Taylor L., Thomson R.J., Nolan P.M., Foster R.G., Peirson S.N. (2017). Meta-analysis of transcriptomic datasets identifies genes enriched in the mammalian circadian pacemaker. Nucleic Acids Res..

[bib12] Brown L.A., Peirson S.N. (2018). Improving reproducibility and candidate selection in transcriptomics using meta-analysis. J. Exp. Neurosci..

[bib15] Cespuglio R., Amrouni D., Meiller A., Buguet A., Gautier-Sauvigné S. (2012). Nitric oxide in the regulation of the sleep-wake states. Sleep Med. Rev..

[bib16] Cheng H.-Y.M., Dziema H., Papp J., Mathur D.P., Koletar M., Ralph M.R., Penninger J.M., Obrietan K. (2006). The molecular gatekeeper Dexras1 sculpts the photic responsiveness of the mammalian circadian clock. J. Neurosci..

[bib17] Cheng H.-Y.M., Obrietan K., Cain S.W., Lee B.Y., Agostino P.V., Joza N.A., Harrington M.E., Ralph M.R., Penninger J.M. (2004). Dexras1 potentiates photic and suppresses nonphotic responses of the circadian clock. Neuron.

[bib18] Choi J.K., Yu U., Kim S., Yoo O.J. (2003). Combining multiple microarray studies and modeling interstudy variation. Bioinformatics.

[bib19] Cismowski M.J., Ma C., Ribas C., Xie X., Spruyt M., Lizano J.S., Lanier S.M., Duzic E. (2000). Activation of heterotrimeric G-protein signaling by a ras-related protein. J. Biol. Chem..

[bib20] Cismowski M.J., Takesono A., Ma C., Lizano J.S., Xie X., Fuernkranz H., Lanier S.M., Duzic E. (1999). Genetic screens in yeast to identify mammalian nonreceptor modulators of G-protein signaling. Nat. Biotechnol..

[bib21] Conti B., Maier R., Barr A.M., Morale M.C., Lu X., Sanna P.P., Bilbe G., Hoyer D., Bartfai T. (2006). Region-specific transcriptional changes following the three antidepressant treatments electro convulsive therapy, sleep deprivation and fluoxetine. Mol. Psychiatr..

[bib22] Dashti H.S., Jones S.E., Wood A.R., Lane J.M., van Hees V.T., Wang H., Rhodes J.A., Song Y., Patel K., Anderson S.G., Beaumont R.N., Bechtold D.A., Bowden J., Cade B.E., Garaulet M., Kyle S.D., Little M.A., Loudon A.S., Luik A.I., Scheer F.A.J.L., Spiegelhalder K., Tyrrell J., Gottlieb D.J., Tiemeier H., Ray D.W., Purcell S.M., Frayling T.M., Redline S., Lawlor D.A., Rutter M.K., Weedon M.N., Saxena R. (2019). Genome-wide association study identifies genetic loci for self-reported habitual sleep duration supported by accelerometer-derived estimates. Nat. Commun..

[bib23] De Leersnyder H., de Blois M.-C., Claustrat B., Romana S., Albrecht U., von Kleist-Retzow J.-C., Delobel B., Viot G., Lyonnet S., Vekemans M., Munnich A. (2001). Inversion of the circadian rhythm of melatonin in the Smith-Magenis syndrome. J. Pediatr..

[bib24] Doherty A., Smith-Byrne K., Ferreira T., Holmes M.V., Holmes C., Pulit S.L., Lindgren C.M. (2018). GWAS identifies 14 loci for device-measured physical activity and sleep duration. Nat. Commun..

[bib25] Duffield G.E. (2003). DNA microarray analyses of circadian timing: the genomic basis of biological time. J. Neuroendocrinol..

[bib26] Edgar R., Domrachev M., Lash A.E. (2002). Gene Expression Omnibus: NCBI gene expression and hybridization array data repository. Nucleic Acids Res..

[bib27] Eglen S.J., Marwick B., Halchenko Y.O., Hanke M., Sufi S., Gleeson P., Silver R.A., Davison A.P., Lanyon L., Abrams M., Wachtler T., Willshaw D.J., Pouzat C., Poline J.-B. (2017). Toward standard practices for sharing computer code and programs in neuroscience. Nat. Neurosci..

[bib28] Elliott A.S., Huber J.D., O'Callaghan J.P., Rosen C.L., Miller D.B. (2014). A review of sleep deprivation studies evaluating the brain transcriptome. SpringerPlus.

[bib29] Fang M., Jaffrey S.R., Sawa A., Ye K., Luo X., Snyder S.H. (2000). Dexras1: a G protein specifically coupled to neuronal nitric oxide synthase via CAPON. Neuron.

[bib30] Fisher R.A. (1935). The logic of inductive inference. J. Roy. Stat. Soc..

[bib31] Graham T.E., Prossnitz E.R., Dorin R.I. (2002). Dexras1/AGS-1 inhibits signal transduction from the Gi-coupled formyl peptide receptor to Erk-1/2 MAP kinases. J. Biol. Chem..

[bib32] Graham T.E., Qiao Z., Dorin R.I. (2004). Dexras1 inhibits adenylyl cyclase. Biochem. Biophys. Res. Commun..

[bib33] Hardin P.E., Panda S. (2013). Circadian timekeeping and output mechanisms in animals. Curr. Opin. Neurobiol..

[bib34] Harrison L.M., He Y. (2011). Rhes and AGS1/Dexras1 affect signaling by dopamine D1 receptors through adenylyl cyclase. J. Neurosci. Res..

[bib35] Hasan S., van der Veen D.R., Winsky-Sommerer R., Hogben A., Laing E.E., Koentgen F., Dijk D.-J., Archer S.N. (2014). A human sleep homeostasis phenotype in mice expressing a primate-specific PER3 variable-number tandem-repeat coding-region polymorphism. FASEB J..

[bib36] Hinard V., Mikhail C., Pradervand S., Curie T., Houtkooper R.H., Auwerx J., Franken P., Tafti M. (2012). Key electrophysiological, molecular, and metabolic signatures of sleep and wakefulness revealed in primary cortical cultures. J. Neurosci..

[bib37] Hoekstra M.M., Emmenegger Y., Hubbard J., Franken P. (2019). Cold-inducible RNA-binding protein (CIRBP) adjusts clock-gene expression and REM-sleep recovery following sleep deprivation. eLife.

[bib38] Jagannath A., Varga N., Dallmann R., Rando G., Gosselin P., Ebrahimjee F., Taylor L., Mosneagu D., Stefaniak J., Walsh S., Palumaa T., Di Pretoro S., Sanghani H., Wakaf Z., Churchill G.C., Galione A., Peirson S.N., Boison D., Brown S.A., Foster R.G., Vasudevan S.R. (2021). Adenosine integrates light and sleep signalling for the regulation of circadian timing in mice. Nat. Commun..

[bib39] Jan M., Gobet N., Diessler S., Franken P., Xenarios I. (2019). A multi-omics digital research object for the genetics of sleep regulation. Sci. Data.

[bib40] Jones S.E., Tyrrell J., Wood A.R., Beaumont R.N., Ruth K.S., Tuke M.A., Yaghootkar H., Hu Y., Teder-Laving M., Hayward C., Roenneberg T., Wilson J.F., Del Greco F., Hicks A.A., Shin C., Yun C.-H., Lee S.K., Metspalu A., Byrne E.M., Gehrman P.R., Tiemeier H., Allebrandt K.V., Freathy R.M., Murray A., Hinds D.A., Frayling T.M., Weedon M.N. (2016). Genome-Wide Association analyses in 128,266 individuals identifies new morningness and sleep duration loci. PLoS Genet..

[bib41] Jones S.E., van Hees V.T., Mazzotti D.R., Marques-Vidal P., Sabia S., van der Spek A., Dashti H.S., Engmann J., Kocevska D., Tyrrell J., Beaumont R.N., Hillsdon M., Ruth K.S., Tuke M.A., Yaghootkar H., Sharp S.A., Ji Y., Harrison J.W., Freathy R.M., Murray A., Luik A.I., Amin N., Lane J.M., Saxena R., Rutter M.K., Tiemeier H., Kutalik Z., Kumari M., Frayling T.M., Weedon M.N., Gehrman P.R., Wood A.R. (2019). Genetic studies of accelerometer-based sleep measures yield new insights into human sleep behaviour. Nat. Commun..

[bib42] Keegan K.P., Pradhan S., Wang J.-P., Allada R. (2007). Meta-analysis of Drosophila circadian microarray studies identifies a novel set of rhythmically expressed genes. PLoS Comput. Biol..

[bib43] Kemppainen R.J., Behrend E.N. (1998). Dexamethasone rapidly induces a novel ras superfamily member-related gene in AtT-20 cells. J. Biol. Chem..

[bib44] Lane J.M., Jones S.E., Dashti H.S., Wood A.R., Aragam K.G., van Hees V.T., Strand L.B., Winsvold B.S., Wang H., Bowden J., Song Y., Patel K., Anderson S.G., Beaumont R.N., Bechtold D.A., Cade B.E., Haas M., Kathiresan S., Little M.A., Luik A.I., Loudon A.S., Purcell S., Richmond R.C., Scheer F.A.J.L., Schormair B., Tyrrell J., Winkelman J.W., Winkelmann J., Sleep H.A.I., Hveem K., Zhao C., Nielsen J.B., Willer C.J., Redline S., Spiegelhalder K., Kyle S.D., Ray D.W., Zwart J.-A., Brumpton B., Frayling T.M., Lawlor D.A., Rutter M.K., Weedon M.N., Saxena R. (2019). Biological and clinical insights from genetics of insomnia symptoms. Nat. Genet..

[bib45] Lane J.M., Liang J., Vlasac I., Anderson S.G., Bechtold D.A., Bowden J., Emsley R., Gill S., Little M.A., Luik A.I., Loudon A., Scheer F.A.J.L., Purcell S.M., Kyle S.D., Lawlor D.A., Zhu X., Redline S., Ray D.W., Rutter M.K., Saxena R. (2017). Genome-wide association analyses of sleep disturbance traits identify new loci and highlight shared genetics with neuropsychiatric and metabolic traits. Nat. Genet..

[bib46] Longordo F., Fan J., Steimer T., Kopp C., Lüthi A. (2011). Do mice habituate to “gentle handling?” A comparison of resting behavior, corticosterone levels and synaptic function in handled and undisturbed C57BL/6J mice. Sleep.

[bib47] Mackiewicz M., Shockley K.R., Romer M.A., Galante R.J., Zimmerman J.E., Naidoo N., Baldwin D.A., Jensen S.T., Churchill G.A., Pack A.I. (2007). Macromolecule biosynthesis: a key function of sleep. Physiol. Genom..

[bib48] Mackiewicz M., Zimmerman J.E., Shockley K.R., Churchill G.A., Pack A.I. (2009). What are microarrays teaching us about sleep?. Trends Mol. Med..

[bib49] Maret S., Dorsaz S., Gurcel L., Pradervand S., Petit B., Pfister C., Hagenbuchle O., O'Hara B.F., Franken P., Tafti M. (2007). Homer1a is a core brain molecular correlate of sleep loss. Proc. Natl. Acad. Sci. USA.

[bib50] Mikhail C., Vaucher A., Jimenez S., Tafti M. (2017). ERK signaling pathway regulates sleep duration through activity-induced gene expression during wakefulness. Sci. Signal..

[bib51] Millius A., Ueda H.R. (2017). Systems biology-derived discoveries of intrinsic clocks. Front. Neurol..

[bib52] Möller-Levet C.S., Archer S.N., Bucca G., Laing E.E., Slak A., Kabiljo R., Lo J.C.Y., Santhi N., von Schantz M., Smith C.P., Dijk D.-J. (2013). Effects of insufficient sleep on circadian rhythmicity and expression amplitude of the human blood transcriptome. Proc. Natl. Acad. Sci. USA.

[bib53] Noya S.B., Colameo D., Brüning F., Spinnler A., Mircsof D., Opitz L., Mann M., Tyagarajan S.K., Robles M.S., Brown S.A. (2019). The forebrain synaptic transcriptome is organized by clocks but its proteome is driven by sleep. Science.

[bib54] Peirson S.N., Butler J.N., Foster R.G. (2003). Experimental validation of novel and conventional approaches to quantitative real-time PCR data analysis. Nucleic Acids Res..

[bib55] Porter N.M., Bohannon J.H., Curran-Rauhut M., Buechel H.M., Dowling A.L.S., Brewer L.D., Popovic J., Thibault V., Kraner S.D., Chen K.C., Blalock E.M. (2012). Hippocampal CA1 transcriptional profile of sleep deprivation: relation to aging and stress. PLoS One.

[bib56] Ramasamy A., Mondry A., Holmes C.C., Altman D.G. (2008). Key issues in conducting a meta-analysis of gene expression microarray datasets. PLoS Med..

[bib57] Shaw P.J., Cirelli C., Greenspan R.J., Tononi G. (2000). Correlates of sleep and waking in *Drosophila melanogaster*. Science.

[bib58] Shen H. (2014). Interactive notebooks: sharing the code. Nature.

[bib59] Song C., Tseng G.C. (2014). Hypothesis SETTING and order statistic for robust genomic meta-analysis. Ann. Appl. Stat..

[bib60] Takesono A., Nowak M.W., Cismowski M., Duzic E., Lanier S.M. (2002). Activator of G-protein signaling 1 blocks GIRK channel activation by a G-protein-coupled receptor. J. Biol. Chem..

[bib61] Tam V., Patel N., Turcotte M., Bossé Y., Paré G., Meyre D. (2019). Benefits and limitations of genome-wide association studies. Nat. Rev. Genet..

[bib62] Team R.C. (2021).

[bib63] Terao A., Steininger T.L., Hyder K., Apte-Deshpande A., Ding J., Rishipathak D., Davis R.W., Heller H.C., Kilduff T.S. (2003). Differential increase in the expression of heat shock protein family members during sleep deprivation and during sleep. Neuroscience.

[bib64] Terao A., Wisor J.P., Peyron C., Apte-Deshpande A., Wurts S.W., Edgar D.M., Kilduff T.S. (2006). Gene expression in the rat brain during sleep deprivation and recovery sleep: an Affymetrix GeneChip study. Neuroscience.

[bib65] Thomas P.D., Ebert D., Muruganujan A., Mushayahama T., Albou L.-P., Mi H. (2022). PANTHER: making genome-scale phylogenetics accessible to all. Protein Sci..

[bib66] Thompson C.L., Wisor J.P., Lee C.-K., Pathak S.D., Gerashchenko D., Smith K.A., Fischer S.R., Kuan C.L., Sunkin S.M., Ng L.L., Lau C., Hawrylycz M., Jones A.R., Kilduff T.S., Lein E.S. (2010). Molecular and anatomical signatures of sleep deprivation in the mouse brain. Front. Neurosci..

[bib67] Uhlén M., Fagerberg L., Hallström B.M., Lindskog C., Oksvold P., Mardinoglu A., Sivertsson Å., Kampf C., Sjöstedt E., Asplund A., Olsson I., Edlund K., Lundberg E., Navani S., Szigyarto C.A.-K., Odeberg J., Djureinovic D., Takanen J.O., Hober S., Alm T., Edqvist P.-H., Berling H., Tegel H., Mulder J., Rockberg J., Nilsson P., Schwenk J.M., Hamsten M., von Feilitzen K., Forsberg M., Persson L., Johansson F., Zwahlen M., von Heijne G., Nielsen J., Pontén F. (2015). Tissue-based map of the human proteome. Science.

[bib68] Vandesompele J., De Preter K., Pattyn F., Poppe B., Van Roy N., De Paepe A., Speleman F. (2002). Accurate normalization of real-time quantitative RT-PCR data by geometric averaging of multiple internal control genes. Genome Biol..

[bib69] Vecsey C.G., Peixoto L., Choi J.H.K., Wimmer M., Jaganath D., Hernandez P.J., Blackwell J., Meda K., Park A.J., Hannenhalli S., Abel T. (2012). Genomic analysis of sleep deprivation reveals translational regulation in the hippocampus. Physiol. Genom..

[bib70] Wang H., Lane J.M., Jones S.E., Dashti H.S., Ollila H.M., Wood A.R., van Hees V.T., Brumpton B., Winsvold B.S., Kantojärvi K., Palviainen T., Cade B.E., Sofer T., Song Y., Patel K., Anderson S.G., Bechtold D.A., Bowden J., Emsley R., Kyle S.D., Little M.A., Loudon A.S., Scheer F.A.J.L., Purcell S.M., Richmond R.C., Spiegelhalder K., Tyrrell J., Zhu X., Hublin C., Kaprio J.A., Kristiansson K., Sulkava S., Paunio T., Hveem K., Nielsen J.B., Willer C.J., Zwart J.-A., Strand L.B., Frayling T.M., Ray D., Lawlor D.A., Rutter M.K., Weedon M.N., Redline S., Saxena R. (2019). Genome-wide association analysis of self-reported daytime sleepiness identifies 42 loci that suggest biological subtypes. Nat. Commun..

[bib71] Williams S.R., Zies D., Mullegama S.V., Grotewiel M.S., Elsea S.H. (2012). Smith-Magenis syndrome results in disruption of CLOCK gene transcription and reveals an integral role for RAI1 in the maintenance of circadian rhythmicity. Am. J. Hum. Genet..

[bib72] Wimmer M.E., Rising J., Galante R.J., Wyner A., Pack A.I., Abel T. (2013). Aging in mice reduces the ability to sustain sleep/wake states. PLoS One.

[bib73] Winrow C.J., Tanis K.Q., Rigby A.M., Taylor R.R., Serikawa K., McWhorter M., Tokiwa G.Y., Marton M.J., Stone D.J., Koblan K.S., Renger J.J. (2009). Refined anatomical isolation of functional sleep circuits exhibits distinctive regional and circadian gene transcriptional profiles. Brain Res..

[bib74] Xu C., Tachmazidou I., Walter K., Ciampi A., Zeggini E., Greenwood C.M.T., Consortium U. (2014). Estimating genome-wide significance for whole-genome sequencing studies. Genet. Epidemiol..

[bib75] Yu G., Wang L.-G., Han Y., He Q.-Y. (2012). clusterProfiler: an R package for comparing biological themes among gene clusters. OMICS.

