# Supplementary material for: Molecular correlates of sleep deprivation in the mouse brain identified by meta-analysis of microarray data
Source: Neurobiol Sleep Circadian Rhythms. 2026 Jun 23;21:100149. doi: 10.1016/j.nbscr.2026.100149 (PMC13382439; doi:10.1016/j.nbscr.2026.100149)
Supplement: Multimedia component 11 [file mmc11.docx]

**Figure S1.** **Baseline behavioural sleep phenotyping in** ***Rasd1* KO mice, shown for males and females.** Baseline behavioural sleep phenotyping in *Rasd1* KO mice. Data pooled into one-hour bins averaged over 7 sequential days of 12:12 LD (left) or 6-hour bins over 7 days (right). Data are shown separately for 9 males (**A-C**) and 9 females (**D-F**) in each genotype. (A,D) Average proportion of sleep, (B,E) average number of bouts, and (C,F) average length of sleep bouts in minutes. Values represent mean ± SEM. Statistical analyses for all behavioural measures are provided in Data Set 10. ***p ≤ 0.001; **p ≤ 0.01; *p < 0.05

**Figure S2.** **Sleep deprivation in *Rasd1* KO mice, combined for males and females.** Effects of sleep deprivation in *Rasd1* KO mice. Data are pooled into six-hour bins. Baseline points are averaged over 7 sequential days of 12:12 LD (left). Animals were sleep deprived for six hours beginning from ZT0 and recorded for four subsequent days (left). Data are combined for both sexes, with 9 males and 9 females of each genotype, representing 18 wild-type and 18 *Rasd1* KO mice. Bar graphs (right) show the immediate hours following sleep deprivation, relative to baseline. (A) Average proportion of behaviourally defined sleep, (B) average number of bouts, and (C) average length of sleep bouts in minutes. Values represent mean ± SEM. Statistical analyses for all behavioural measures are provided in Data Set 10.

**Figure S3.** **Baseline hourly activity in *Rasd1* KO mice.** Baseline behavioural activity phenotyping in *Rasd1* KO mice. Data is pooled into one-hour bins averaged over 7 sequential days of 12:12 LD. Data are shown for both sexes combined in (A) representing 18 wild-type and 18 *Rasd1* KO mice, and separately for each sex in (B) and (C) representing 9 wild-type and 9 *Rasd1* KO mice. Values represent mean ± SEM. Statistical analyses for all behavioural measures are provided in Data Set 10. ***p ≤ 0.001; **p ≤ 0.01; *p < 0.05
