# Supplementary material for: Molecular correlates of sleep deprivation in the mouse brain identified by meta-analysis of microarray data
Source: Neurobiol Sleep Circadian Rhythms. 2026 Jun 23;21:100149. doi: 10.1016/j.nbscr.2026.100149 (PMC13382439; doi:10.1016/j.nbscr.2026.100149)
Supplement: Multimedia component 12 [file mmc12.docx]

**Table S1. Comparison of known sleep genes from previous studies (Elliott et al., 2014)**

**against genes of interest identified in this study.** Measures of combined effect size (M*) as well as heterogeneity across 23 pairwise comparisons (Hedges G SD and consistency%). Known sleep genes show larger combined effect sizes (M*) and high consistency in the direction of change across the 23 pairwise comparisons making up the meta-analysis. By contrast, the 8 genes of interest characterised in this study showed smaller but reproducible effect sizes. These effects are expected in a meta-analysis, which is able to detect smaller but reproducible changes across multiple studies. M* Rank: Ranked combined effect size (M*), from 1 (greatest effect size) to 498 (smallest effect size). Hedges G SD Rank: Ranked standard deviation of effect size across 23 pairwise comparisons (Hedges g), from 1 (most reproducible across 23 pairwise comparisons) to 498 (least reproducible). Consistency%: Proportion of 23 pairwise comparisons showing same direction of change (up or down regulated), expressed as percentage.

| **Group** | **Gene** | **M* Rank** | **Hedges G SD Rank** | **Consistency%** |
| --- | --- | --- | --- | --- |
| **19 known sleep genes** | *Arc* | 26 | 472 | 96% |
|  | *Bdnf* | 20 | 448 | 96% |
|  | *Dbp* | 492 | 477 | 100% |
|  | *Egr1* | 36 | 484 | 91% |
|  | *Egr2* | 13 | 332 | 100% |
|  | *Fos* | 23 | 394 | 96% |
|  | *Cirbp* | 493 | 467 | 100% |
|  | *Creld2* | 2 | 466 | 100% |
|  | *Crem* | 18 | 252 | 100% |
|  | *Homer1* | 68 | 468 | 91% |
|  | *Hspa1b* | 9 | 403 | 96% |
|  | *Hspa5* | 1 | 490 | 100% |
|  | *Nr4a1* | 22 | 384 | 91% |
|  | *Per2* | 80 | 407 | 100% |
|  | *Rbm3* | 495 | 495 | 100% |
|  | *Sytl2* | 491 | 471 | 100% |
|  | *Usp2* | 463 | 417 | 83% |
|  | *Vip* | 95 | 330 | 83% |
|  | *Xbp1* | 12 | 470 | 100% |
|  |  |  |  |  |
| **8 genes of interest** | *Arntl* | 57 | 363 | 91% |
|  | *Fastkd5* | 92 | 224 | 96% |
|  | *Hsf1* | 214 | 343 | 91% |
|  | *Mag* | 275 | 245 | 96% |
|  | *Maoa* | 371 | 453 | 96% |
|  | *Naglu* | 397 | 171 | 87% |
|  | *Rasd1* | 195 | 162 | 83% |
|  | *Tipin* | 471 | 462 | 91% |
