# Supplementary material for: Molecular correlates of sleep deprivation in the mouse brain identified by meta-analysis of microarray data
Source: Neurobiol Sleep Circadian Rhythms. 2026 Jun 23;21:100149. doi: 10.1016/j.nbscr.2026.100149 (PMC13382439; doi:10.1016/j.nbscr.2026.100149)
Supplement: Multimedia component 13 [file mmc13.docx]

***Molecular correlates of sleep deprivation in the mouse brain identified by meta-analysis of microarray data***

**USER GUIDE TO SUPPLEMENTARY DATA SETS**

The following document provides a simple overview of the data sets accompanying this manuscript. This includes a summary of all the supplementary data sets accompanying the main manuscript and a description of what each data set contains. There are then a set of example questions relating to how users can use the published data as a resource and apply this to their own research.

**Description of Supplementary Data Sets**

**Data Set S1**. Summary data for all initial listings, including those subsequently excluded. This includes publication, strain, sex and age of mice/rats used, duration of sleep deprivation, method of sleep deprivation, tissues collected, collection time (ZT) and microarray platform used.

**Data Set S2**. Justifications for all study exclusions. Includes publication, PubMed ID, inclusion/exclusion status and reasons for exclusion.

**Data Set S3**. Successfully mapped genes for comparison with human GWAS data. This includes the human orthologs of the 498 significant genes, organised into up- (194 genes) and down-regulated (243 genes). These orthologs were the basis of the PEGS analysis to compare against human GWAS data.

**Data Set S4**. PEGS results. The number of the 498 significant genes from the sleep deprivation meta-analysis that fall within peak sets of defined distances from human sleep/circadian GWAS loci. Intervals used were: 5kbp, 50kbp, 100kbp, 500kbp, 1000kbp and 2000kbp. Different GWAS studies and traits were used, including chronotype, daytime sleepiness, insomnia, long duration and short duration sleep. The number of up- and down-regulated genes within each interval are shown in the ‘common genes’ sheet and the p-values of enrichment are shown in the ‘P values’ sheet.

**Data Set S5**. Individual comparisons of 498 significant genes. This includes the 498 significant genes, with results for the 23 pairwise comparisons that make up the meta-analysis. Headings for the individual 23 pairwise comparisons are shown in the Appendix to this user guide for ease. Sheets include the effect size in the 23 pairwise comparisons (Hedges g), Z values, p-values, a summary of all calculations, meta-analysis weighted results and a key of the summaries and calculations used.

**Data Set S6**. Summary of all 498 significant genes. This is the main summary of the significant genes identified in this meta-analysis, replicating the meta-analysis weighted results in Data Set S5.. This sheet includes for each gene name, the combined effect size (M*), Random Effects Model SEM, Z value, P-value and Q-value (FDR corrected p-value), the log10 Q-value, Ensembl gene id and description. Multiple identifiers are used to prevent any confusion with the recognised problems of autocorrection of gene names in tools such as MS Excel. This is presented as a pivot-table to allow easy sorting of data by each column.

**Data Set S7**. Results sheets including M* and q value data for all 28,220 genes. This sheet presents the same data as Data Set S7, but for all 28,220 genes irrespective of whether they are significant. This can be used to evaluate whether genes that were not classified as significant in this meta-analysis were significant is one or more of the 23 pairwise comparisons. Headings for the individual 23 pairwise comparisons are shown in the Appendix to this user guide.

**Data Set S8**. Gene lists for significant intervals. The up- and down-regulated genes from this meta-analysis that were significantly enriched for different human GWAS traits related to sleep, including insomnia, long duration and short duration sleep. Different peak set intervals are shown.

**Data Set S9**. Short duration up-regulated genes Gene Ontology. The Gene Ontology enrichment for short duration sleep upregulated genes, using the Panther over-representation tool. Only the gene list from the Short Duration SNPs with an interval of 2Mb showed significant pathway enrichment for processes within the GO biological process (complete) dataset.

**Data Set S10**. Additional analysis of Rasd1KO mice behavioural data. This includes sleep proportion, number of bouts, length of bouts both at baseline (BL) and following sleep deprivation (DEP). This also includes activity data at baseline (BL). Where sex differences were observed, grouped as well as male and female data are also provided.

**Using the data in this study as a resource**

To provide some examples as to how the data included in this manuscript can be used a resource for other researchers in this field, a series of questions are presented below, with answers directing the user to where to locate the relevant data in the supplementary data sets.

1. ***Which microarray data sets are included in the analysis?*** The data sets used in the meta-analysis are summarised in the manuscript in **Table 1**. The individual studies considered are shown in Data Set S1, and inclusion and exclusion criteria are shown in Data Set S2.
2. ***Which genes are most robustly affected by sleep deprivation across studies?*** The 498 genes that are significantly different (Q<0.01) are shown in Data Set S6. The combined effect size is shown as M*, which gives a combined measure of how robust effect sizes are across the 23 pairwise comparisons.
3. ***Which genes show robust changes but show high heterogeneity across studies?*** The meta-analysis is based on 23 pairwise comparisons of data sets from 6 publications. These specific comparisons are summarised in Table 1 in the main manuscript, and the coding used in the supplementary data sets is provided in a the Appendix of this guide. To generate a Forest plot for any gene, the individual effect size (Hedges g) can be plotted across the 23 pairwise comparisons. These individual effect sizes are shown in Data Set S5, under the Hedges g sheet for all 496 significant genes. To provide an indication of the heterogeneity of the data, the standard deviation of the Hedges g statistic is shown at the far right, highlighted in blue. This is also ranked from 1 (lowest variance) to 498 (highest). Another metric, “consistency%” is provided, which classifies the proportion of the 23 pairwise comparisons that show changes in the same direction (up-regulated vs down-regulated). 100% indicates that all 23 pairwise comparisons show changes in the same direction.
4. ***Which genes are cortex-specific versus robust across all studies?*** To determine if changes are cortex specific in comparison with hypothalamus, oligodendrocytes, astrocytes, hippocampus or whole brain, the Hedges g statistic can be compared for the first 5 columns in Data Set S5 (MoEx_CerCx, 430AV2_CerCx_3h, 430AV2_CerCx_6h, 430AV2_CerCx_9h, 430AV2_CerCx_12h) against other columns. This approach can be used to identify whether significant genes in the meta-analysis are changing more or less in any particular tissue or cell compartment included in the data.
5. ***My gene of interest isn’t differentially expressed, but can I find how it changes in response to sleep deprivation?*** If not included in the main 498 significant genes in the meta-analysis provided in Data Set S6, researchers can explore Data Set S7, which includes data for all 28,220 genes included. This can be used to determine if a particular gene shows changes in any of the 23 pairwise comparisons or falls just outside the q<0.01 false detection rate used in this meta-analysis.
6. ***How can I identify genes that are supported by human genetic data?*** Not all mouse genes may map to human genes, so of the 498 sleep DEGs, the successfully human mapped up- and down-regulated DEGs are shown in Data Set S3. The interval around known GWAS hits for specific traits are summarised in Data Set S4 with the number of genes within each interval. The specific genes within these intervals are summarised in Data Set in S8.

**Appendix**

Key to column coding in Data Set S5 and Data Set S7.

| **Code prefix** | **Platform** | **GSE** | **Publication** | **PubMed ID** |
| --- | --- | --- | --- | --- |
| MoEx_CerCx | Affymetrix Mouse Exon Array | SE33491 | Hinard et al., 2012 | 22956841 |
| 430AV2_CerCx_3h | Affymetrix 430 Array Version 2.0 | GSE6514 | Mackiewicz et al. 2007 | 17698924 |
| 430AV2_CerCx_6h | Affymetrix 430 Array Version 2.0 | GSE6514 | Mackiewicz et al. 2007 | 17698924 |
| 430AV2_CerCx_9h | Affymetrix 430 Array Version 2.0 | GSE6514 | Mackiewicz et al. 2007 | 17698924 |
| 430AV2_CerCx_12h | Affymetrix 430 Array Version 2.0 | GSE6514 | Mackiewicz et al. 2007 | 17698924 |
| 430AV2_HypoT_3h | Affymetrix 430 Array Version 2.0 | GSE6514 | Mackiewicz et al. 2007 | 17698924 |
| 430AV2_HypoT_6h | Affymetrix 430 Array Version 2.0 | GSE6514 | Mackiewicz et al. 2007 | 17698924 |
| 430AV2_HypoT_9h | Affymetrix 430 Array Version 2.0 | GSE6514 | Mackiewicz et al. 2007 | 17698924 |
| 430AV2_HypoT_12h | Affymetrix 430 Array Version 2.0 | GSE6514 | Mackiewicz et al. 2007 | 17698924 |
| 430AV2_ForeNonOlig | Affymetrix 430 Array Version 2.0 | GSE48369 | Bellesi et al. 2013 | 24005282 |
| 430AV2_ForeOlig | Affymetrix 430 Array Version 2.0 | GSE48369 | Bellesi et al. 2013 | 24005282 |
| 430AV2_Hipp | Affymetrix 430 Array Version 2.0 | GSE33302 | Vecsey et al., 2012 | 22930738 |
| 430AV2_Astro | Affymetrix 430 Array Version 2.0 | GSE69079 | Bellesi et al. 2015 | 26413480 |
| 430AV2_NonAstro | Affymetrix 430 Array Version 2.0 | GSE69079 | Bellesi et al. 2015 | 26413480 |
| 430AV2_WB_AK | Affymetrix 430 Array Version 2.0 | GSE9441 | Maret et al. 2007 | 18077435 |
| 430AV2_WB_B6 | Affymetrix 430 Array Version 2.0 | GSE9441 | Maret et al. 2007 | 18077435 |
| 430AV2_WB_D2 | Affymetrix 430 Array Version 2.0 | GSE9441 | Maret et al. 2007 | 18077435 |
| 430AV2_ZT12_AK | Affymetrix 430 Array Version 2.0 | GSE9441 | Maret et al. 2007 | 18077435 |
| 430AV2_ZT12_B6 | Affymetrix 430 Array Version 2.0 | GSE9441 | Maret et al. 2007 | 18077435 |
| 430AV2_ZT12_D2 | Affymetrix 430 Array Version 2.0 | GSE9441 | Maret et al. 2007 | 18077435 |
| 430AV2_ZT6_AK | Affymetrix 430 Array Version 2.0 | GSE9441 | Maret et al. 2007 | 18077435 |
| 430AV2_ZT6_B6 | Affymetrix 430 Array Version 2.0 | GSE9441 | Maret et al. 2007 | 18077435 |
| 430AV2_ZT6_D2 | Affymetrix 430 Array Version 2.0 | GSE9441 | Maret et al. 2007 | 18077435 |
